# Experimental and numerical investigation of single-slope solar still performance enhanced by porous absorbing materials: thermal, economic, and environmental assessments

**DOI:** 10.1038/s41598-026-41901-9

**Published:** 2026-03-07

**Authors:** Sajjad Hameed Majeed, Farhan Lafta Rashid, Haider Nadhom Azziz, Arman Ameen

**Affiliations:** 1 Mechanical Engineering Department/College of Engineering, University of Kerbala, Karbala, 56001 Iraq; 2 Petroleum Engineering Department/College of Engineering, University of Kerbala, Karbala, 56001 Iraq; 3 Department of Building Engineering, Energy Systems and Sustainability Science, University of Gävle, 801 76 Gävle, Sweden

**Keywords:** Solar still, Porous materials, Thermal efficiency, Economic analysis, Environmental assessment, Desalination, Energy science and technology, Engineering, Environmental sciences

## Abstract

Low freshwater productivity and poor thermal efficiency remain key limitations of conventional single-slope solar stills. In this study, porous absorbing materials are investigated as passive performance-enhancement strategies for small-scale solar desalination. A combined experimental and numerical analysis was conducted on a traditional solar still (TSS) and two modified configurations incorporating melamine sponge (MSSS) and pumice stone (VPSSS), operated under real climatic conditions in Karbala, Iraq. The results demonstrate that the MSSS achieved the highest daily freshwater yield of 1347 mL/day, corresponding to a 56.9% increase compared with the TSS, alongside an average thermal efficiency of 49.3%. The VPSSS produced 1055 mL/day, representing a 22.9% improvement and a thermal efficiency of 38.2%. Economic analysis indicates that, under optimal operating conditions, the MSSS reduced the water production cost to 0.07569 USD/L with a payback period of approximately 2.5 years. The energy payback period ranged from 0.55 to 0.86 years, whereas the exergy recovery period remained considerably longer (28–35 years), highlighting inherent thermodynamic limitations. In addition, the MSSS configuration achieved an annual CO_2_ emission reduction of approximately 1612 kg, corresponding to a cost saving of 17.36 USD. Overall, the findings suggest that porous absorbing materials, particularly melamine sponge, offer an effective and economically feasible approach for enhancing solar still performance in arid and remote regions.

## Introduction

The solar desalination technology has proved as one of the viable methods to tackle the world water crisis especially whereby water resources are minimal in the arid and the semi-arid areas. Solar stills are one of the most used and affordable technologies of solar desalination that can be used to generate fresh water by using saline or brackish water. The basic physics of solar stills consists of the greenhouse effect as the sun rays heat water in a basin resulting in evaporation and the steam then condenses onto a colder surface to form a distilled water. Nevertheless, relatively low productivity of the traditional solar stills has induced the researchers to seek to identify several approaches to enhance their thermal efficiency and water yield.

The recent developments in the solar still technology have aimed at optimizing the various design configurations towards the maximization of the productivity. Hussein et al.^1^ made a criterion survey of pyramid solar stills; this has done recent designs, performance characteristics and configurations that have demonstrated promising outputs in the area of maximizing water production. Continuing on this, Kadhim et al.^2^ discussed the innovations in the geometry of the basin, namely, hemispherical solar stills and the potential to enhance productivity caused by the distribution of heat and the process of condensation. The history of the solar still design is marked by an ongoing struggle to compromise the limitation of traditional flat-basin solar still design.

Wick material integration has become one of the major strategies of performance improvement in solar stills. The comparative experimental study by Kadhim et al.^3^ on the use of different wick materials in single-slope solar stills yielded extensive energy, exergy and economic analysis to prove significant freshwater yield improvement. This strategy enhances the evaporative surface area and it promotes better heat transfer and high distillation rates. Moreover, a detailed survey of conical solar stills with internal and external modifications was described by Al-Obaidi et al.^4^, the authors concluded that the various geometries of solar still have had a variety of approaches taken by the researchers to achieve optimum performance of the solar still to suit the desired purpose.

Thermal energy storage methods have been found to be important in lengthening the working hours of solar stills, which are not accessible around the daylight hours. A specific review on how to improve the productivity of hemispherical solar stills with thermal energy storage techniques was given by Kadhim et al.^5^ and it was noted that it is important to keep basin temperatures high during off-sun periods. In addition to this study, a research by Ben Hamida et al.^6^ experimentally investigated the process of improving water yield of the pyramid solar stills with aluminum fins, and showed that metallic fins are capable of enhancing the spread of heat in the system and consequently raising the overall system thermal efficiency.

Porous absorbing materials incorporation is one of the most effective methods of solar stills passive reinforcement. An energy, exergy, and environmental analysis of the solar stills were developed with porous materials along with the saline water pre-heating and solar panel combination provided a background to the multi-faceted advantages of porous absorbers^7^. Ramzy et al.^8^ also examined the performance of single-slope solar stills with various porous absorbing materials based on experimental methods and found out that the choice of material is a major determinant of evaporation rates and general productivity. Wu et al.^9^ conducted a review of thermal absorbers and their integration techniques into solar photovoltaic/thermal modules, which can give insights into the potential of the technology of absorbers to solar desalination.

Different porous substances have also been explored to improve the solar still efficiency. Arunkumar et al.^10^ illustrated the increase in productivity when porous absorbers were used with bubble wrap insulation and it was established that thermal insulation is a complement to ensure the high temperatures in the basement basins. Pal et al.^11^ examined the modified basin-type multi-wick solar stills and found the benefits of the combination of multiple improvement methods. Setareh et al.^12^ conducted experimental studies on stepped solar stills through porous materials and this study performed to help in comprehending the effect of surface area augmentation on the distillation efficiency.

Recent technology has addressed non-traditional methods of enhancement of porous materials. Aghakhani et al.^13^ made comparisons between classic configurations of wire mesh and vibrating wire mesh and proved that the mechanical vibration with porous material can lead to significant performance improvement. Sathyamurthy^14^ experimentally studied solar stills with porous rubber sheets, as storage energy material, and demonstrated that porous materials with elastic characteristics can be used in two applications. Dumka et al.^15^ studied the incorporation of the Plexiglas and jute cloth with an investigation of the natural fiber-based porous materials as a sustainable alternative. Setareh et al.^16^ also performed additional tests on the stepped solar stills using porous material, which confirms previous results of the effectiveness of the solar stills.

Nanotechnology and specialized coating have also been included in advanced enhancement technique. A techno-enviro-exergo-economic analysis of non-cover box solar stills using parabolic dish concentrators and thermoelectric Peltier effects was conducted by Nazari and Daghigh^17^. In a pioneering effort in the use of nanotechnology in solar desalination, Elango et al.^18^ researched on single-basin single-slope solar stills using various water nanofluids. Mahian et al.^19^ examined the role of nanofluids on the rate of evaporation in solar stills that were installed with heat exchangers. The articles by Satyamurthy et al.^20^ and Panchal et al.^21^ experimentally studied the efficacy of the use of MgO and TiO_2_ nanoparticles in stepped solar stills and manganese oxide nanoparticle-coated absorbers respectively. Arani et al.^22^ investigated the effect of using fins and silicon dioxide nanoparticle black paint on the absorber plate. The study by Kabeel et al.^23^ revealed the impact of the water depth on the pyramid solar still absorber plates covered with TiO_2_ nano black paint. Sharshir et al.^24^ examined the various materials of heat localization and Thakur and Sathyamurthy^25^ did their research using the various plates that were coated with microparticles and gave a detailed yield, thermal, economic and environmental analysis.

Among other things, novel materials have been investigated to enhance the absorber plates. Abdelal and Taamneh^26^ explored carbon fiber/CNT modified epoxy composite to use on the plates of the pyramid solar still absorbers. Mehta et al.^27^ were interested in enhancing the solar still efficiencies with optimized fin geometry and affordable selective coating. Parikh et al.^28^ experimentally tested the enhancement of productivity with the help of hybrid nanomaterials and water-cooling. The study by Bekele et al.^29^ compared the modified passive solar stills with nanoparticle coating and nanoparticle-enhanced phase change material. Pradhan et al.^30^ came up with ferrite nano-coating in fast and sustainable desalination. The study by Bansal et al.^31^ involved the validation and CFD simulation of solar stills with nanoparticle coating on the absorber plate. Panchal et al.^32^ studied the graphite powder add with black paint on absorber plates to increase yield. Madhua et al.^33^ made a comparison of conventional and stepped solar stills with various nanofluids.

The entire performance analysis should take into account several evaluation standards. Yousef and Hassan^34^ carried out vigorous and physical performance appraisals of inclusion of phase change material on solar distillation systems. Hedayati-Mehdiabadi et al.^35^ compared the performance of basin-type phase-change materials and PV/T collectors in solar stills to exergy performance. Ahmadi et al.^36^ conducted analysis of energy, exergy and the environmental aspects of combined heat and power systems which can be used in solar desalination as they presented methodological frameworks. Yousef and Hassan^37^ studied the energy payback time, exergoeconomic, and enviroeconomic of thermal energy storage systems which have solar desalination. Exergoeconomic, enviroeconomic, and productivity analyses of basin-type solar stills using PVT compound parabolic concentrator collectors were done by Singh and Tiwari^38^. Shatar et al.^39^ conducted energy, exergy, economic and environmental analyses of solar stills through partially coated condensing covers that have thermoelectric cooling. Khalaf et al.^40^ conducted a thorough overview of available solar still technologies and cost evaluating the innovations in materials, design, and methods to increase the efficiency of water desalination.

Dhivagar et al.^41^ evaluated a new strategy where a solar photovoltaic (SPV) panel is placed on the other side of a double-slope solar still (DSS) with A46 phase change material (PCM) to improve the energy conversion, thermal management, and freshwater production to achieve the dual advantage of electricity and distillate whereby the PCM leveled the variation in temperature and the system increased its productivity cumulatively by 25.6% and 8.8% and 9.1% in energy and exergy efficiency Dhivagar et al.^42^ evaluated thermal characteristics of a heat pump assisted regenerative solar still (HPARSS) with slack wax heat storage medium, in which thermal energy provided by condensation was recovered through a compression heat pump to pre-heat the inlet saline water, they recorded that the HPARSS had maximum cumulative productivity of 16.5 kg/m^2^ (85.1% more than a conventional solar still (CSS)) and helped significantly in using less energy and exergy 67.4% and 81.9% better A modified double-slope solar still (MDSS) was presented by Dhivagar et al.^43^ that mounted a solar PV panel directly on top, allowing trigeneration of electricity, heat, and freshwater, which showed 71.4%, 39%, and 41.9% higher cumulative productivity, 39% and 51.4% higher energy efficiency and exergy efficiency respectively, and 11.1%, 10.1% lower cost-per-liter (CPL) of distillate and payback period (PBP) A new DSS with paraffin wax as a heat store material to achieve efficient cogeneration in order to have an effective SPV panel and paraffin wax on a single side was introduced by Dhivagar et al.^44^ which revealed that the SPV-DSS was 42.3%, 41.5%, and 155.9% times more productive, energy efficient, and exergy efficient in a day than a traditional DSS with a 45.3% and 7.5% energy and exergy efficiency, respectively. Dhivagar et al.^45^ compared the use of beeswax and paraffin-based PCMs in a full 5E framework in solar still desalination systems, and indicated that a system with beeswax-integrated system (BWSS) was 8.7%, 6.7%, and 6.1% times more productive, efficient, and exergetic than a system with paraffin wax-integrated system (PWSS). It is also lowering production cost by 7.7% and payback period by 4.9% as well as increasing profit-cost ratio by 7.9%.

Boka et al.^46^ considered two strategies: developing a cloud-based IoT system with remote monitoring and data acquisition and increasing the performance by incorporating toner waste powder nanoparticles in black paint at 5% and 10% content, and their IoT platform allowed them to monitor parameters continuously and the coated black paint to achieve higher basin and cover temperatures than the standard one. Sudalaimuthu et al.^47^ adopted the biowick materials with amendments of iron oxide nanoparticles to significantly enhance the freshwater production of inclined solar stills, which were found to experience higher levels of water temperatures and daily yields than when using the pure biowick, and also exhibited higher levels of thermal and exergy efficiencies. Atia et al.^48^ describe the latest advances and point out that their presence in everyday yield and productivity through latent heat storage can be greatly enhanced by phase change materials, and that porous media such as steel wool can also achieve high productivity improvements by enhancing the number of evaporative surfaces. Essa et al.^49^ proposed a full redesign of the traditional spherical solar still, and showed significant performance improvements with a corrugated absorber, jute wicking, preheated feedwater and nocturnal thermal storage which resulted in a maximum productivity increase of 170%. Besides, Essa et al.^50^ increased the productivity of a pyramid solar still by adding a vertically-placed-triangular-shaped absorber with single-axis Solar tracking system and reflectors, with increases of up to 130 in productivity.

Recent solar still developments concentrate on the improvement of productivity using new designs, stage change materials (PCM) as well as nanoparticles. Essa et al.^51^ designed a paraffin wax and graphite and Al_2_O_3_ nanoparticles jute wick hemispherical solar distiller (HSD) that yielded 6370 mL m^–^^2^day^–^^1^ and 48.2% energy efficiency, which enhanced yield with/without thermal storage material (TSM) by 90.74% and 41.73%, respectively. El-Sebaey et al.^52^ upgraded a semi-cylindrical paraffin wax PCM stepped-basins solar still (SCSBSS), achieving 3889 mL m^–2^ day^–1^, a 44.52% improvement, at 20 L saltwater depth. The research of Satyamurthy et al.^53^ enhanced a single slope solar still (SSSS) with carbon soot nanoparticles (50–60 nm) covered on soda cans in PCM, and the experiments showed yield increase of 75.7 and 102.3% compared to non-PCM and uncoated systems. Rajamony et al.^54^ prepared binary Ag and coconut shell biochar (CSB) nanoparticles in A46 PCM, which enhanced thermal conductivity (102.15, 0.23–0.465 W/mK) and EStorage (158.6–167.8 J/G). El-Sebaey et al.^55^ constructed a CFD model to predict solar still without experiment and verified their model at a 1.982 L/m^2^ simulated that was against 1.785 L/m^2^ experimental yield at a 2 cm depth. El-Sebaey et al.^56^ constructed a single-slope two-basin still, where the depth was 2 cm, and 2.855 L/m^–^^2^ day^–1^, a 59.9% increase in yield and a 61.3% increase in thermal efficiency was obtained compared to conventional. Mevada et al.^57^ combined a zig-zag air-cooled condenser (ZZACC) and CuO nanoparticles to enhance distillate by 46.83% and daily energy efficiency by 45.98%. Essa et al.^58^ optimized a rotating spherical solar distiller (RBSD) at 0.5 rpm, wick-preheated at 65 °C, and achieved 6200 mL/m^–^^2^ day^–1^, which is 91% times higher than traditional.

The main unique aspect of the study consists in the combined experimental and numerical study of the single-slope solar stills with two different porous substances, i.e. melamine sponge and pumice stone, under the actual climatic conditions in Karbala, Iraq, with the combined multi-criteria evaluation related to the thermal, economic, energy, exergy and environmental aspects. The main objectives are to measure and compare the performance enhancement of freshwater productivity, thermal efficiency, and cost-effectiveness of performance of the traditional still with the two modified designs and to compare numerical simulations of ANSYS Fluent with experimental observations and to undertake holistic analyses of performance; energy payback time, exergy recovery, life cycle conversion efficiency, and CO_2_ emission avoidance of using these porous absorbers in small-scale solar desalination in dry areas.

This work is novel because it uses a combined experimental and numerical study of single slope solar stills, which are improved with two different yet low cost porous materials to the melamine sponge and pumice stone under authentic climatic conditions in Karbala, Iraq. With a multi-criteria evaluation of thermal performance, economic viability, energy-exergy efficiency, and environmental impact in a single framework, unlike the prior investigations that involved single enhancement methods or ideal environments, this study offers a multi-criteria evaluation. The integrated approach of empirical validation, manipulation of computational fluid dynamics (CFD) through ANSYS Fluent, and sustainability metrics on holistic basis is a new and viable avenue to optimization of small-scale solar desalination in arid regions, which directly solves the issues of material-specific performance comparison and applicability.

## Experimental setup

The study aims at performing experimental upgrading of a single-slope solar still by incorporating porous absorbing substances in the basin. Two substances were testable and compared against a conventional still with no porous media, which are melamine sponge and pumice stone. The experiments were carried out on Natural sunlight on September 16, 2025 in Karbala, Iraq (32.6167^o^N and 44.0333^o^E). Water surface temperature, vapor temperature, and glass cover temperature, as well as daily productivity were key performance indicators, which were systematically measured and analysed to determine the effect of each of the porous materials on the solar still performance. The K-type thermocouples (calibrated 0.5 °C) were used to measure the temperature, solar irradiance was measured with a pyranometer (0.5 W/m^2^), digital hygrometer (± 0.1 °C, ± 0.1% RH) was used to measure the ambient temperature and humidity, and the wind speed was measured using an anemometer. This was filled with brackish water at a depth of 2 cm salinity level of 3500 ppm.

**Fig. 1:**
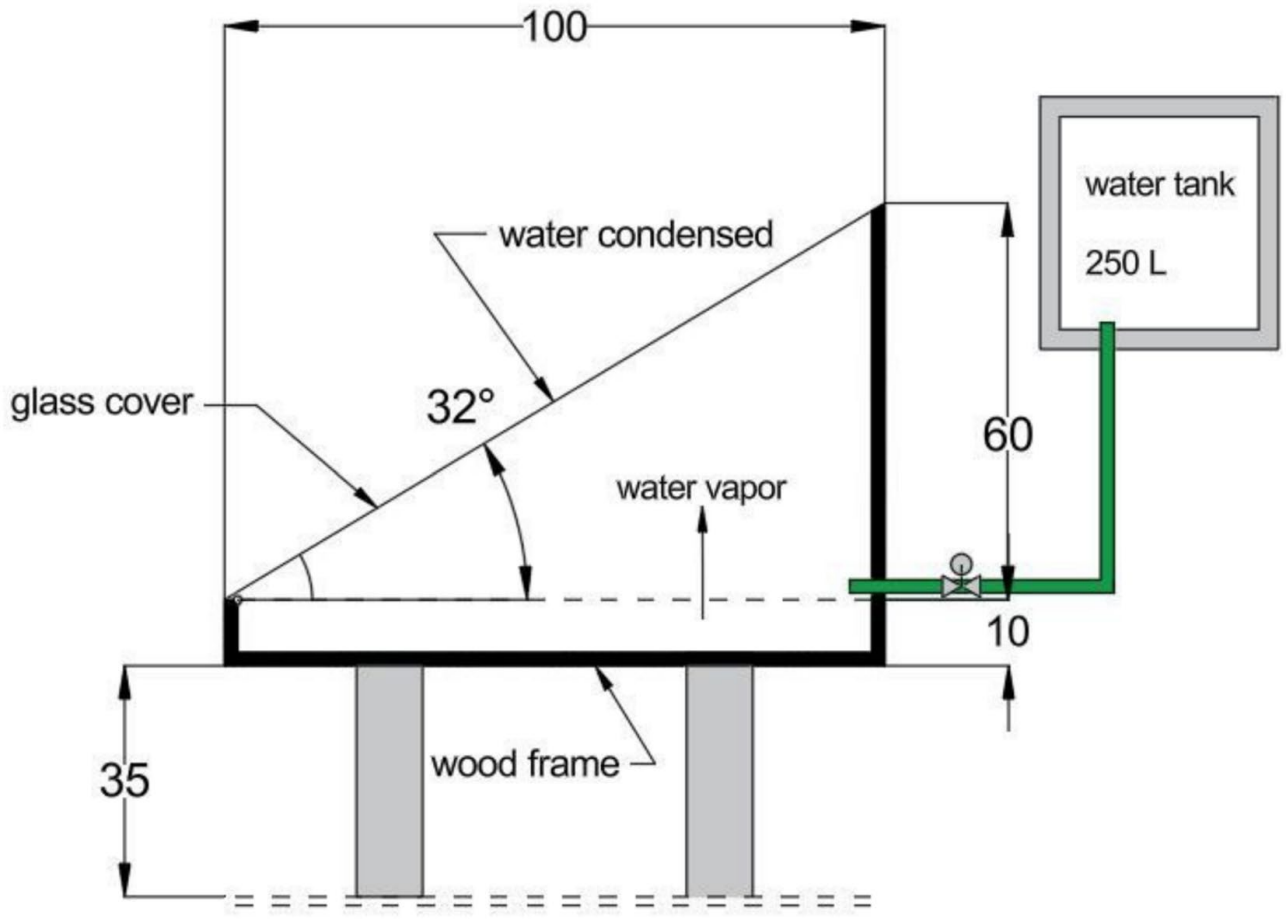
Schematic diagram of the experimental setup [Adapted from 8].

Figure 1 schematically illustrates the experimental configuration of the single-slope solar still, where the main parts are illustrated which are the inclined glass cover, insulated basin, collection ports of saline and distilled water in and out, and the integration points of the porous absorbing materials (melamine sponge and pumice stone). The diagram gives a good illustration of the geometry of the system and how the solar radiation goes through the glass cover to the saline water in the basin, to heat it then the evaporation and condensation process takes place to yield fresh water. It is an effective scheme that grounds the experimental setup and facilitates the latter thermal and performance analysis presented within the framework of the research.

**Fig. 2:**
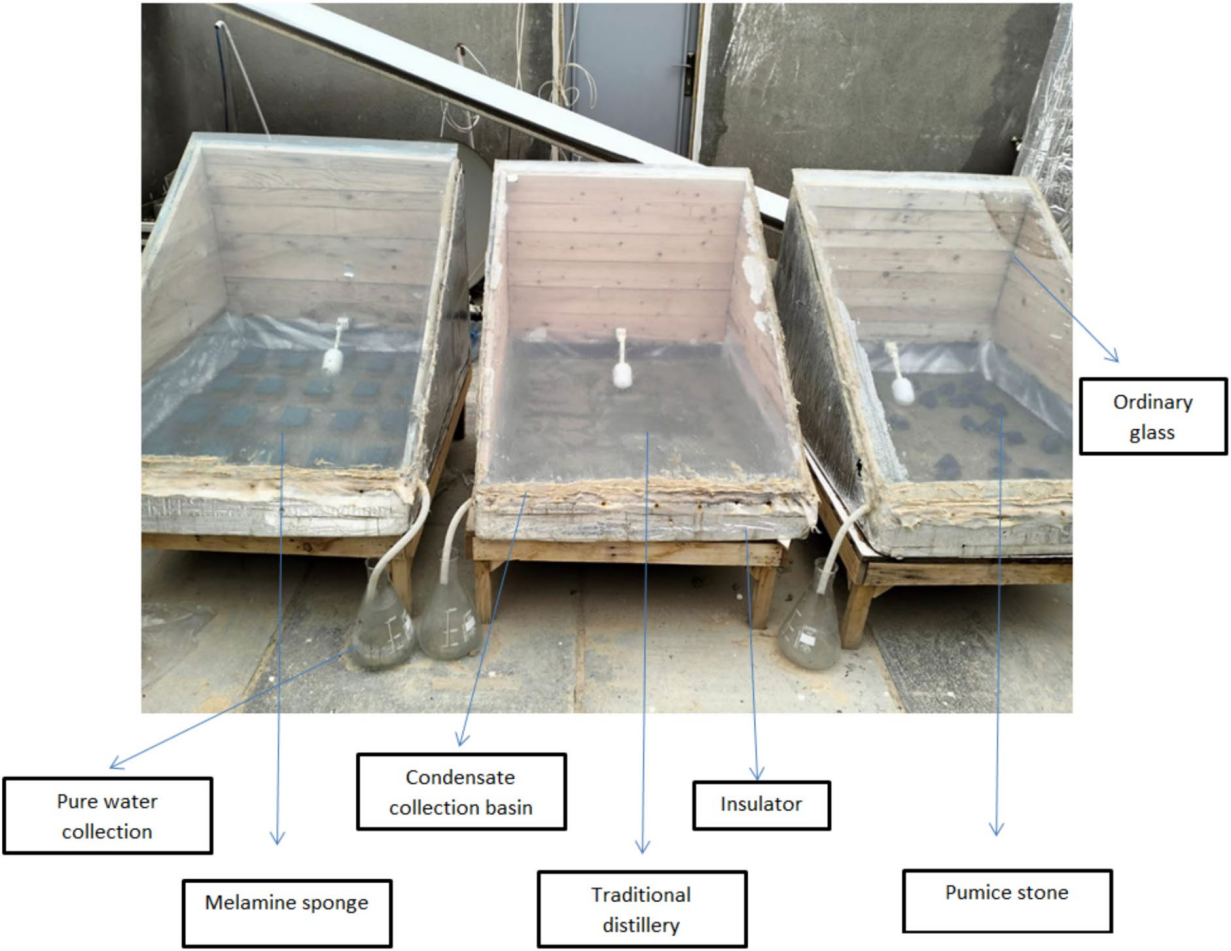
Photographic view of solar still basin for the three cases.

A photo-Comparison of the three solar still arrangements studied such as the Traditional Solar Still (TSS), the pumice stone-enhanced still (VPSSS), and the melamine sponge-enhanced still (MSSS) are given in Fig. 2. The pictures well depict the physical layout and composition of the porous substances in the basin, which puts emphasis on the structural divergences of the set-ups. The melamine sponge is represented as a light and porous filling that occupies the basin and the pumice stone is represented as a denser and granular material. The TSS basin, which is free of porous media, forms the basis of visual and performance comparison, which will highlight the modifications made to the basin to improve the evaporation and thermal retention in the two enhanced designs.

Table 1 also compared the main thermophysical characteristics of melamine sponge and pumice stone, which indicate their unique features. The thermal conductivity of the melamine sponge is very low (0.035 W/mK) with very high porosity (> 99%) and very low density (about 8 kg/m^3^). On the other hand, pumice stone has better thermal conductivity (approximately 0.15 W/mK), medium porosity (about 65%), and increased density (about 500 kg/m^3^) which increases its time cooling capacity as well as its capillary action. These variations underscore the enhancement dissimilarities: the sponge maximizes the evaporation performance whereas the pumice stone enhances the retention of heat and the distribution of water inside the solar still.

**Table 1:** Thermophysical properties of the porous absorbing materials^59,60^.

Property	Melamine sponge	Pumice stone
Thermal Conductivity	Very Low (~ 0.035 W/mK)	Low (~ 0.15 W/mK)
Porosity	Extremely High (> 99%)	High (~ 65%)
Density	Very Low (~ 8 kg/m^3^)	Low (~ 500 kg/m^3^)

Experimental apparatus employed K-type thermocouples to measure temperature (± 0.5 °C), Kipp and Zonen CMP6 pyranometer to measure sun irradiance (± 2%), and a graduated cylinder to collect distillate (± 5 mL). In order to determine the aggregate uncertainty in the experimental results, the root-sum-square method was used. Given a result R, which is a function of n independent variables × 1, × 2, etc. i.e. R = f(× 1, × 2, …, xn), the joint uncertainty UR is given by:

$${U}_{R}=\sqrt{{\left(\frac{\partial R}{\partial x1}{U}_{x1}\right)}^{2}+{\left(\frac{\partial R}{\partial x2}{U}_{x2}\right)}^{2}+{\left(\frac{\partial R}{\partial x3}{U}_{x3}\right)}^{2}}$$

A brief overview of the tools utilized in data collection is given in Table 2, which lists all the parameters measured using the tools, as well as, types, accuracies and the estimated uncertainties therein. This is a methodical overview that provides clarity concerning reliability and reproducibility of the measurements in the experimental research.

**Table 2:** Summary of measurement instruments and estimated uncertainties.

Parameter measured	Instrument used	Accuracy/tolerance	Estimated uncertainty
Solar irradiance	Kipp & Zonen CMP6 Pyranometer	± 2%	± 2%
Temperature (Water, Vapor, Glass)	K-type thermocouple	± 0.5 °C	± 1.1% (at 45 °C)
Distillate volume	Graduated cylinder	± 5 mL	± 2.5% (for 200 mL)
Daily productivity	Derived from volume & Area	-	± 2.8%

## Mathematical formulation

### Cost evaluation analysis

Economic analysis is meant to state the price of manufacturing a Liter of water^21,30,33^.

- Capital Recovery factor CRF

It is based on this factor to make the initial cost of investment to be transformed into a fixed cost of a year of operation.

1 $$CRF=\frac{i(i+1{)}^{z}}{\left[(i+1{)}^{z}-1\right]} \, (\frac{1}{year})$$

i Interest rate and taking to be 10% (0.1).

z Number of years of operation (z = 10 year).

- Fixed annual cost (FAC)

This is the overall cost of the distillery that is represented as an annual expenditure in the course of operation.

2 $${\mathrm{FAC}}\, = \,{\mathrm{PCC}}.{\text{CRF }}\left( {\$ /{\mathrm{year}}} \right)$$

PCC is Primary Capital cost ($) and represent the amount paid to set up the solar still.

- Annual salvage value (ASV)

The amount of salvage value that the system will have at the end of its life every year. It transforms the salvage value into a yearly value.

3 $${\mathrm{ASV}}\, = \,{\mathrm{S}}.{\text{SFF }}\left( {\$ /{\mathrm{Year}}} \right)$$

Where:

## Sinking funded factor (SFF)

It is any variable, like CRF, based on which the annual salvage value is determined in the case when the system reaches the expiration of the useful life.

4 $$SFF=\frac{i}{\left[{\left(i+1\right)}^{z}-1\right]}\left(\frac{1}{year}\right)$$

Annual salvage (S)

The value of the amount of distillate cost is what one gets when it is sold as salvage at the expiry of its usefulness. The value of the salvage is considered to be 20% of the original capital cost.

5 $${\mathrm{S}}\, = \,0.{2 }\left( {{\mathrm{PCC}}} \right) \, \left( \$ \right)$$

- Annual maintenance cost (AMC)

The glass cleaning, salt and limescale removal, water refilling and small maintenance are all operating and maintenance costs of 15% of the Fixed annual cost (FAC).

6 $${\mathrm{AMC}}\, = \,0.{15}.{\mathrm{FAC}}\left( {\$ /{\mathrm{year}}} \right)$$

- Annual cost (AC)

The sum of the system, operation, and maintenance cost, minus the cost of the salvage, and is calculated as an annual value during the operating life.

7 $$AC = FAC + AMC - ASV\left( {\$ /{\mathrm{year}}} \right)$$

- Average annual productivity (AAP)

The quantity of water produced per year is the number of operating days of a year multiplied by the daily production.

AAP = the number of operating days per year x daily production (Liter/Year).

${\dot{m}}_{w}$= total mass of distilled water produced per day (Liter/day).

number of operating days per year = 340 (day/Year).

- Cost per Liter CPL

This represents the cost of producing one Liter.

8 $$CPL=\frac{AC}{AAP} \, (\frac{\$}{Lit})$$

- Payback period (n_p_)

It is the period of years or days that the net profits of the sale of distilled water will cover the cost of the establishment of the system. System cost = Present value of profit generated cash flows during the payback period.

9 $$PCC={\sum }_{t=1}^{np}({\mathit{CF}}_{t}(i+1{)}^{-np}$$

where $C{F}_{t}$ is year net cash flow.

If the cash flow is constant at that time, then:

10 $$\begin{gathered} CF = CF_{t} \\ PCC = CF \cdot FRP_{i,n} \end{gathered}$$

where FRP (Factor recovery period) is the recovery period factor, which takes into account the annual interest rate during the recovery period.

11 $$FRP_{i,n} = \frac{{(1 + i)^{np} - 1}}{{i(1 + i)^{np} }}\,\,\,n_{p} = \ln (\frac{CF}{{CF - PCC}})/\ln (1 + i){ }\left( {{\mathrm{Year}}} \right)$$

To calculate CF:

($/year) CF = Average Annual Productivity (AAP) (Liter/year) x Selling price per Liter (S/Liter).

Where selling price per Liter is taken to be from 0.12 to 0.24 S/Liter.

### Energy matrices

**EPBT (Energy payback time)**

It is the energy recovery time and is the figure of time used by the system to generate useful energy similar to the energy used in the process of it being manufactured or created^23–25^.

12 $$(EPBT{)}_{en}=\frac{E_{in}}{{E}_{annual,out}} ({\mathrm{Year}})$$

where *E*_*in*_ represents the total energy required to manufacture and assemble the distillate materials and is calculated as follows:

13 $$E_{in}=\sum {m}_{i}.{e}_{i} \, ({\mathrm{MJ}})$$

${e}_{i}$ is the specific energy embodied in a material or used in its manufacture (Mj/Kg).

${m}_{i}$ is the mass of substance i (kg).

14 $$E_{in} = \sum {\left( {m_{glass} e_{glass} + m_{wood} e_{wood} + m_{rubber} e_{rubber} + m_{{pa{\mathrm{int}} }} e_{{pa{\mathrm{int}} }} + m_{additive} e_{additive} } \right)}$$

${E}_{Annual,out}$ is Annual energy produced and represent the Thermal energy used in water evaporation during the year.

15 $$\left(\frac{MJ}{Year}\right){E}_{Annual,out}=AAP\left(\frac{Kg}{Year}\right)\cdot {h}_{fg}\left(\frac{MJ}{Kg}\right)$$

The exact values of energy of the components of the system and the mass of each of them are indicated in Table below.

**Table:** 

Materials	e (MJ/Kg)	mass (Kg)
Wood	10	23.4
Glass	15.9	9.3
Paint	90	2
Rubber	67	1
Addition materials		
TSS	–	––
VPSSS	3	2.52
MSSS	35	0.16

The energy or exergy quantities may take the unit of MJ or kWh, where:

1 kWh = 3.6MJ

The values of EPBT may be compared in terms of energy and exergy. Energy is concerned with how much energy is produced whereas exergy is concerned with how good the energy is and how it is utilized. Energy is founded on latent heat whereas exergy is founded on usable exergy. Exergy is grounded on the idea that not all one Joule of thermal energy can be completely converted into useful work.

16 $$(\mathit{EPBT}{)}_{ex}=\frac{{Ex}_{in}}{{Ex}_{Annual,out}}$$

${Ex}_{in}={E}_{in}$ Because high-quality manufacturing energy

17 $${Ex}_{{Annual,out}}={E}_{Annual,out}\left(1-\frac{{T}_{o}}{{T}_{w}}\right)$$

where T_o_ is the ambient temperature and equal to 298 K, T_W_ is the Water temperature.

### Energy production factor (EPF)

The energy production factor is the number of times that a system generates more energy than the energy used in making it; the reverse of EPBT. This is an indicator of sustainability of energy and does not correlate with the economic factor; the larger the number, the better the system^2^.

18 $$\begin{gathered} EPF_{en} = \frac{E_{Annual,out}}{E_{in}} = \frac{1}{EPBT_{en}} \\EPF_{ex} = \frac{Ex_{Annual,out}}{Ex_{in}} = \frac{1}{EPBT_{ex}} \end{gathered}$$

### Life cycle converts efficiency (LCCE)

Lifecycle conversion efficiency is a measure that describes how efficiently the system can convert the energy obtained by sunlight over the period of its anticipated useful life into net usable energy and the energy devoted to its production. The operating life and manufacturing energy is associated with energy sustainability^2^.

19 $$(\mathit{LCCE}{)}_{en}=\frac{({E}_{annual,out}\cdot Z)-{E}_{in}}{{E}_{solar,total}}$$

${E}_{annual,out}$ total energy produced by the system during the year (MJ/year).

Z is operation life (year).

${E}_{solar,total}$ Total solar energy incident on the system during its operating life

20 $${E}_{solar,total}={I}_{ar}\cdot A\cdot {t}_{sun}\cdot 3600\cdot 340\cdot Z\cdot 1{0}^{-6} \, ({\mathrm{MJ}})$$

The quantity ${t}_{sun}\cdot 3600\cdot 340\cdot Z$ represent the operating hours during the system’s lifetime (Z) and are measured in (sec).

${t}_{sun}$ is the number of hours of sunshine per day (h/day).

${I}_{ar}$ Average solar radiation intensity during sunshine hours (W/m^2^).

A Distiller surface area (m^2^).

Z Operating life (Year).

As for the $(\mathit{LCCE}{)}_{ex}$, it shows the ratio of net usable exergy produced during the operating lifetime compared to the incident solar exergy during the system’s lifetime. It is a metric that measures conversion quality.

21 $$(\mathit{LCCE}{)}_{ex}=\frac{({\mathit{Ex}}_{\mathit{annual},\mathit{out}} \cdot Z)-{Ex}_{in}}{E{x}_{solar,total}}$$

$E{x}_{solar,total}$ is the total exergy falling from the sun during the lifespan of the system.

22 $$Ex_{solar,total} = E_{solar,total} (1 - \frac{4}{3}\cdot\frac{T_o}{T_S} + \frac{1}{3}(\frac{T_o}{T_S})^4)$$

${T}_{S}$ Sun surface temperature (5777 K).

### Exergy-economic analysis

The analysis is what connects exergy losses in the system to the economic cost i.e. where the exergy is lost and the cost of this loss. It is often associated with enhancement of the destructive exergy reduction and not only growth of the power^7,36^.

23 $$\begin{gathered} C_{ex} = \frac{AC(\$ /year)}{{E_{{x_{annual,out} }} (kWh/year)}} = \frac{AC}{{(E_{{x_{annual,out} }} \left( {MJ)/3.6} \right)}} \left( {\frac{\$ }{kWh}} \right) \hfill \\ C_{en} = \frac{AC}{{E_{annual,out} (kWh/year)}} = \frac{AC}{{(E_{annual,out} \left( {MJ)/3.6} \right)}} \left( {\frac{\$ }{kWh}} \right) \hfill \\ \end{gathered}$$

The above quantity represents the cost of producing one unit of exergy or energy.

24 $$R_{ex} = \frac{1}{{C_{ex} }}{ }\left( {\frac{kWh}{\$ }} \right)\,\,R_{en} = \frac{1}{{C_{en} }}{ }\left( {\frac{kWh}{\$ }} \right)$$

The above number is the useful exergy or energy that can be generated by the system per dollar expended on it because the cost of energy does not indicate quality.

### Environmental and economic analysis

The eco-economic analysis of the system should help to evaluate the economic greatness of the environmental advantage^7,36,39^. What is the number of CO_2_ emissions avoided with the solar still? And what is the economic worth of this amount?

The emission factor (CO_2_) of conventional fossil fuel power plants with a thermal efficiency of 0.38 is $0.96\frac{k{g}_{c{o}_{2}}}{kWh}$. This means that 1 kWh of electricity produced from coal generates $0.96\frac{k{g}_{c{o}_{2}}}{kWh}$. Nevertheless, once the power gets to the house, a fifth of this resource goes to the drain through inefficient heaters and outmoded appliances. Moreover, there is a loss of this energy by about 40% in transmission and distribution. Thus, greater than this value of power should be generated to get a useable 1 kWh. In case the total efficiency of the electrical system:

$${\eta }_{{e}_{1}}=(1-0.20)(1-0.40)=0.8\times 0.6=0.48$$

This means that 48% of the energy produced is actually utilized, and therefore the electricity required from the station $\frac{1}{0.48}=2.08(kWh)$ .

Therefore, to obtain a useful 1 kWh, the actual amount of CO_2_ released is:

25 $${m}_{c{o}_{2}}=0.96\left(\frac{k{g}_{c{o}_{2}}}{1kwh}\right)\times 2.08(\frac{kwh}{1kwh})\simeq 2\left(\frac{k{g}_{c{o}_{2}}}{kwh}\right)$$

$${\psi }_{c{o}_{2}}={m}_{c{o}_{2}}=2\left(\frac{kg}{kWh}\right)$$

If the total amount of thermal energy supplied to the distiller (produced by it):

26 $${q}_{in}={E}_{out,annual}\frac{(kWh)}{year}=\frac{{E}_{out,annual}(\mathit{MJ}/\mathit{year})}{3.6}$$

We therefore dispense with the amount of electricity generated by this heat. If the conversion efficiency from electricity to heat is 0.38, then the amount of electricity dispense is $\left(\frac{{E}_{out,annual}}{0.38}\right)$.

Therefore, we reduce the amount of CO_2_ emissions by:

27 $$({\mathit{kg}}_{c{o}_{2}}/\mathit{year}){\varphi }_{c{o}_{2}} =\frac{{E}_{out,annual}}{0.38}\frac{\left(kWh\right)}{Year}\times {\psi }_{c{o}_{2}}\left(\frac{k{g}_{c{o}_{2}}}{kWh}\right)$$

If the cost of treating (CO_2_) is:

28 $${Z}_{c{o}_{2}}=10.76\left(\frac{\$}{ton}\right)$$

The environmental cost savings from using the distiller are:

29 $$\left(\frac{\$}{year}\right){X}_{c{o}_{2}}={Z}_{c{o}_{2}}\left(\frac{\$}{kg}\right){\varphi }_{c{o}_{2}}\left(\frac{kg}{year}\right)$$

### Uncertainty analysis for energy and exergy analysis parameters

The overall uncertainty in energy-related and exergy-related results was evaluated using the root-sum-square method. For a derived parameter *R* dependent on independent variables × 1, × 2,…*, xn*​, the combined uncertainty *UR*​ is expressed as:

30 $${U}_{R}=\sqrt{{\left(\frac{\partial R}{\partial x1}{U}_{x1}\right)}^{2}+{\left(\frac{\partial R}{\partial x2}{U}_{x2}\right)}^{2}+\dots +{\left(\frac{\partial R}{\partial xn}{U}_{xn}\right)}^{2}}$$

Specifically, for daily energy output ${E}={m}^{.}{h}_{fg}$​, where ${m}$ is the distillate mass flow rate and ${h}_{fg}$ is the latent heat, the uncertainty is calculated as:

31 $${U}_{{E}}=\sqrt{{\left({h}_{fg}\cdot{U}_{m}\right)}^{2}+{\left({m}\cdot{U}_{{h}_{fg}}\right)}^{2}}$$

For exergy output ${E}_{x}={E}\left(1-\frac{{T}_{0}}{{T}_{w}}\right)$, the uncertainty is given by:

32 $${U}_{{E}_{x}}=\sqrt{{\left(\left(1-\frac{{T}_{0}}{{T}_{w}}\right){U}_{E}\right)}^{2}+{\left(\frac{{E}{T}_{0}}{{T}_{w}^{2}}{U}_{{T}_{w}}\right)}^{2}}$$

where ${U}_{m}$​, ${U}_{{h}_{fg}}$​​, and ${U}_{{T}_{w}}$​​ are the uncertainties in mass flow, latent heat, and water temperature, respectively.

### Energy and exergy modelling

The evaluations of energy and exergy were part of the comprehensive energy analysis of exergy in this research. The energy modelling measured the overall amount of thermal energy consumption and output, and obtained key performance indicators, including the energy payback time (EPBT), energy production factor (EPF), and life cycle conversion efficiency (LCCE) in an energy basis. At the same time, exergy modelling to measure the quality and useful work potential of the energy flows considering thermodynamic irreversibility was used to give exergy-based EPBT, EPF, and LCCE. These two analyses demonstrated that, although the modified stills greatly enhance the energy recovery (i.e., MSSS EPBTen = 0.55 years) the exergy recovery times are longer (28–35 years), which is a major thermodynamic drawback of low-temperature solar distillation. This combined energy-exergy method offers a more comprehensive and strict evaluation of the thermodynamic functionality of the system, its sustainability and the optimization possibilities.

## Results and discussion

### Thermal analysis

**Fig. 3:**
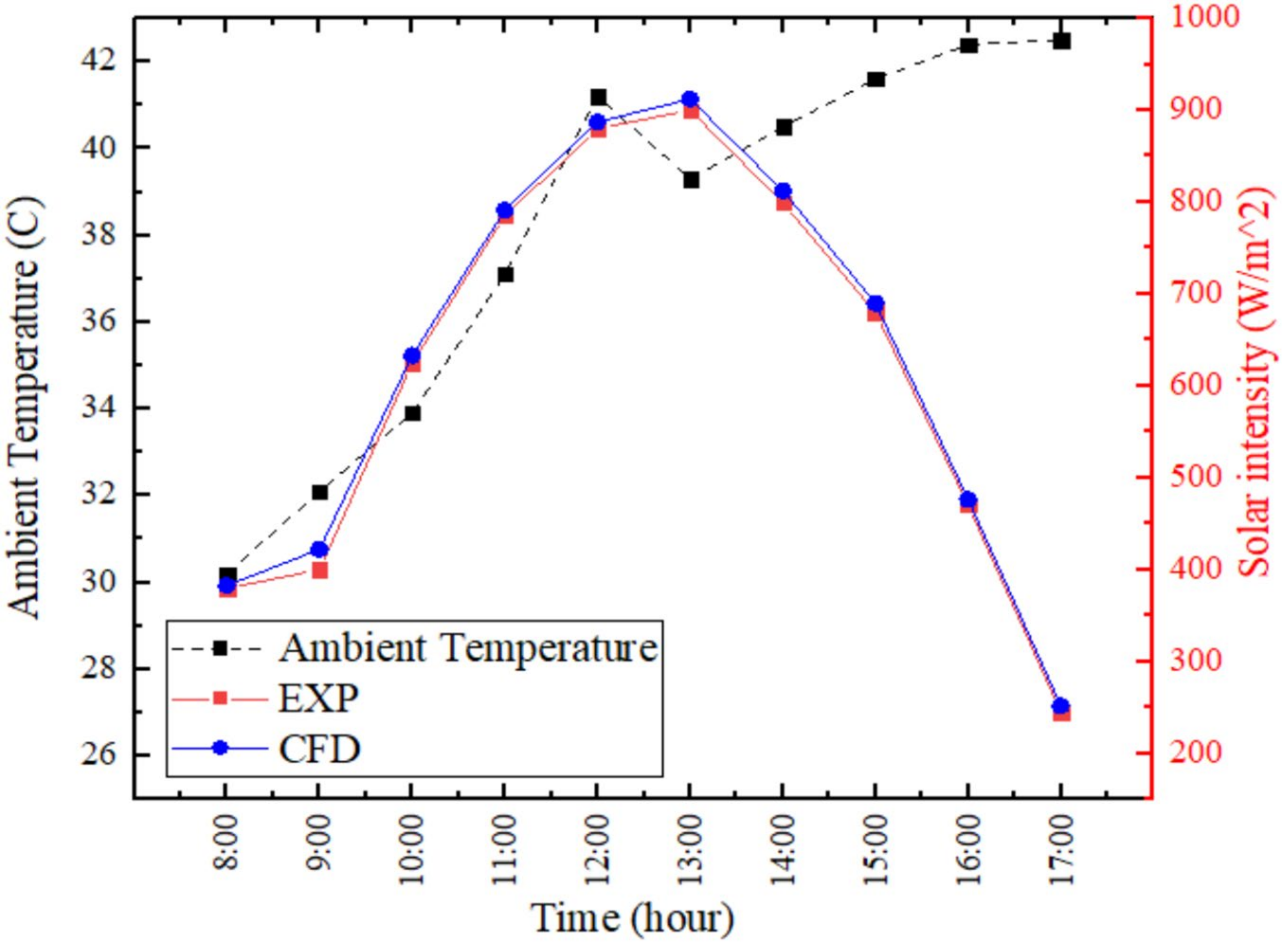
Experimental and numerical variation in solar radiation intensity at the solar still.

According to Fig. 3, the experimental and numerical values of solar radiation are very similar with the maximum irradiance of about 970 W/m^2^ around noon time. Experimental curve has some small variations because of the actual atmospheric changes (e.g. temporary cloud cover or aerosol scattering) whereas the numerical model presumes a smooth idealized profile of the sun. The close match shows that inputs to the simulation are accurate and justifies the use of the numerical model to predict performance. These small variations are caused by the instrument response limits and unrepresented environmental effects, yet the overall pattern suggests the validity of the further thermal and productivity results obtained under the measured solar conditions. The near correspondence between experimental and numerical profiles of solar irradiance is a confirmation of the precision of the input values and boundaries conditions in our ANSYS Fluent model. The small deviations, probably caused by temporal atmospheric attenuation, exist within a reasonable range of uncertainty, and it proves the correctness of the model in further thermal and productivity simulations in the conditions of the measured sun.

**Fig. 4:**
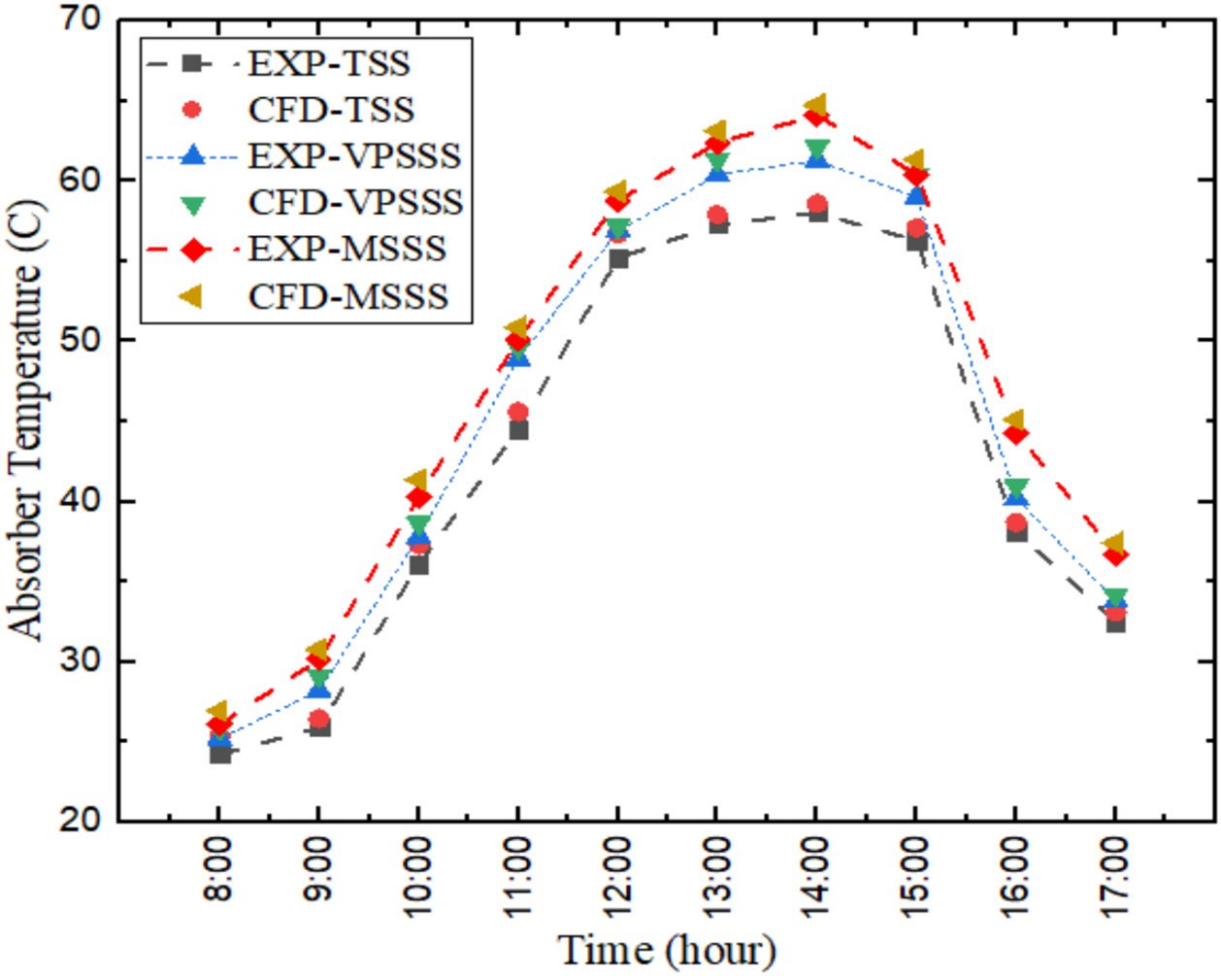
Temperature variation in absorber experimentally and numerically.

Figure 4 indicates that the experimental and numerical values of the absorber temperature variation are very much in agreement and the average relative error is about 2.36%. It is found that the highest temperature of the absorber is around 2:00 PM and it is the highest in the solar still enhanced by melamine sponge (MSSS), then the pumice stone-enhanced still (VPSSS) and the traditional one (TSS). This is physically explained by the increased solar absorption and thermal retention properties of the porous materials: the high porosity of melamine sponge (> 99%), combined with low thermal conductivity (~ 0.035 W/mK) of the material, ensures high rate of heat absorption and local evaporation, whereas the moderate porosity (~ 65%) of the pumice stone, and its higher density (~ 500 kg/m^3^), gives higher thermal mass and sustained heat distribution, respectively, resulting in higher absorber temperatures. The thermal model is confirmed by the agreement between the experimental and numerical absorber temperatures with the best peak temperature (approximately 74 °C ) being the melamine sponge owing to its ultra-high porosity (> 99%) and low thermal conductivity (approximately 0.035 W/m.K) that increases local solar absorption and minimizes conductive losses, to establish a steeper thermal gradient to facilitate evaporation.

**Fig. 5:**
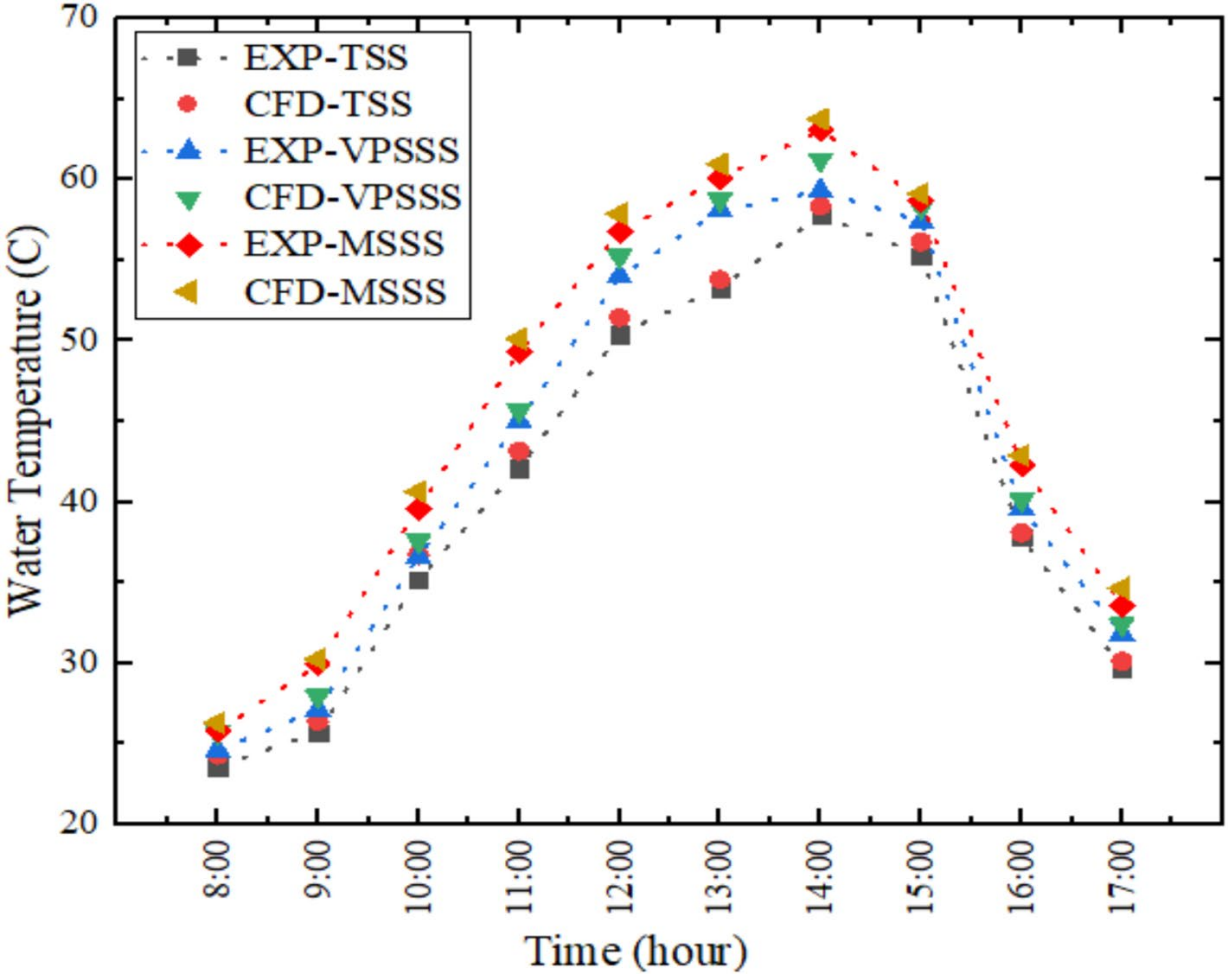
Water temperature variation in basin experimentally and numerically.

Figure 5 indicates that both experimental and numerical values of water temperature change in the basin are in agreement as an average relative error is about 2.36%. The maximum temperature is approximately 2:00 PM 57.8 °C in the traditional still (TPSS), 59.3 °C in the VPSSS (pumice stone-enhanced still), and 63.1 °C in the MSSS (melamine sponge-enhanced still). The increased temperatures in modified stills are attributable to the increased thermal absorption and retention characteristics of the porous materials; the melamine sponge increases the area of evaporation and enables faster evaporation, and the pumice stone enhances the thermal mass and distribution of capillary water, both of which results in a more effective use of solar energy and an increase in the temperature of the water.

**Fig. 6:**
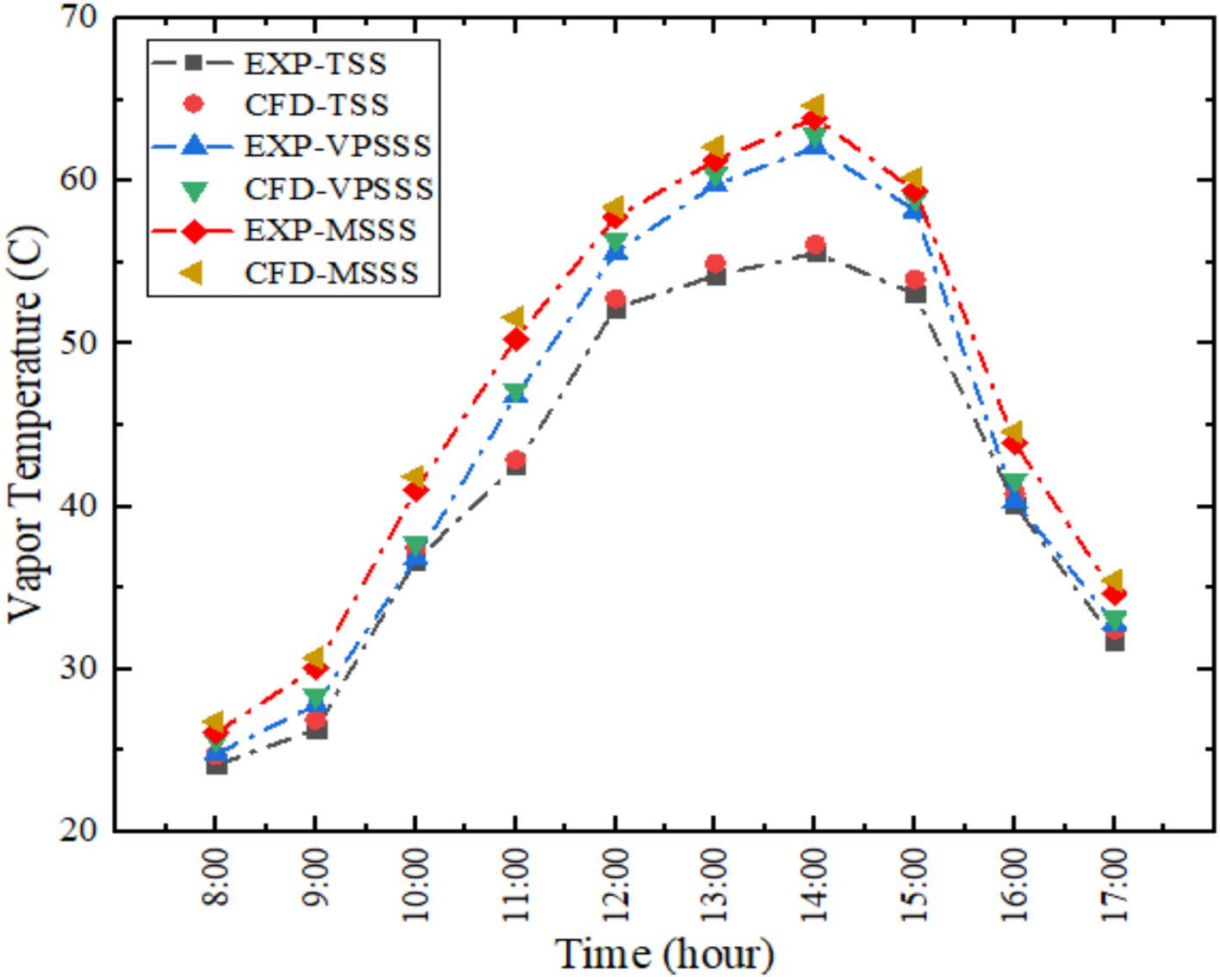
Vapor temperature variation experimentally and numerically.

Figure 6 represents the experimental and numerical variation of the vapor temperature inside the solar still that indicate that there is a high degree of agreement in the measured and simulated data with an average relative error of about 2.36. The vapor temperature shoots up at the hemline of 2:00 PM, and higher in the modified stills, especially in the MSSS (Melamine Sponge) owing to an increase in the evaporation as a result of the porous materials. The high porosity (> 99%) and low density of the melamine sponge also ensure high air–water interface to generate more as much vapour, whereas the moderate porosity and thermal mass of the pumice stone serve to store heat, which causes the high vapour temperatures relative to the more conventional still. This increase of the vapor temperature is directly associated with more productivity because the higher the temperature of the vapor, the higher the rate of condensation on the cooler piece of glass. The thermal-fluid correlation is also confirmed by the close agreement (avg. error = 2.36) as the vapor temperatures in modified stills are directly proportional to the partial pressure and to the rate of evaporation in the presence of porous media.

**Fig. 7:**
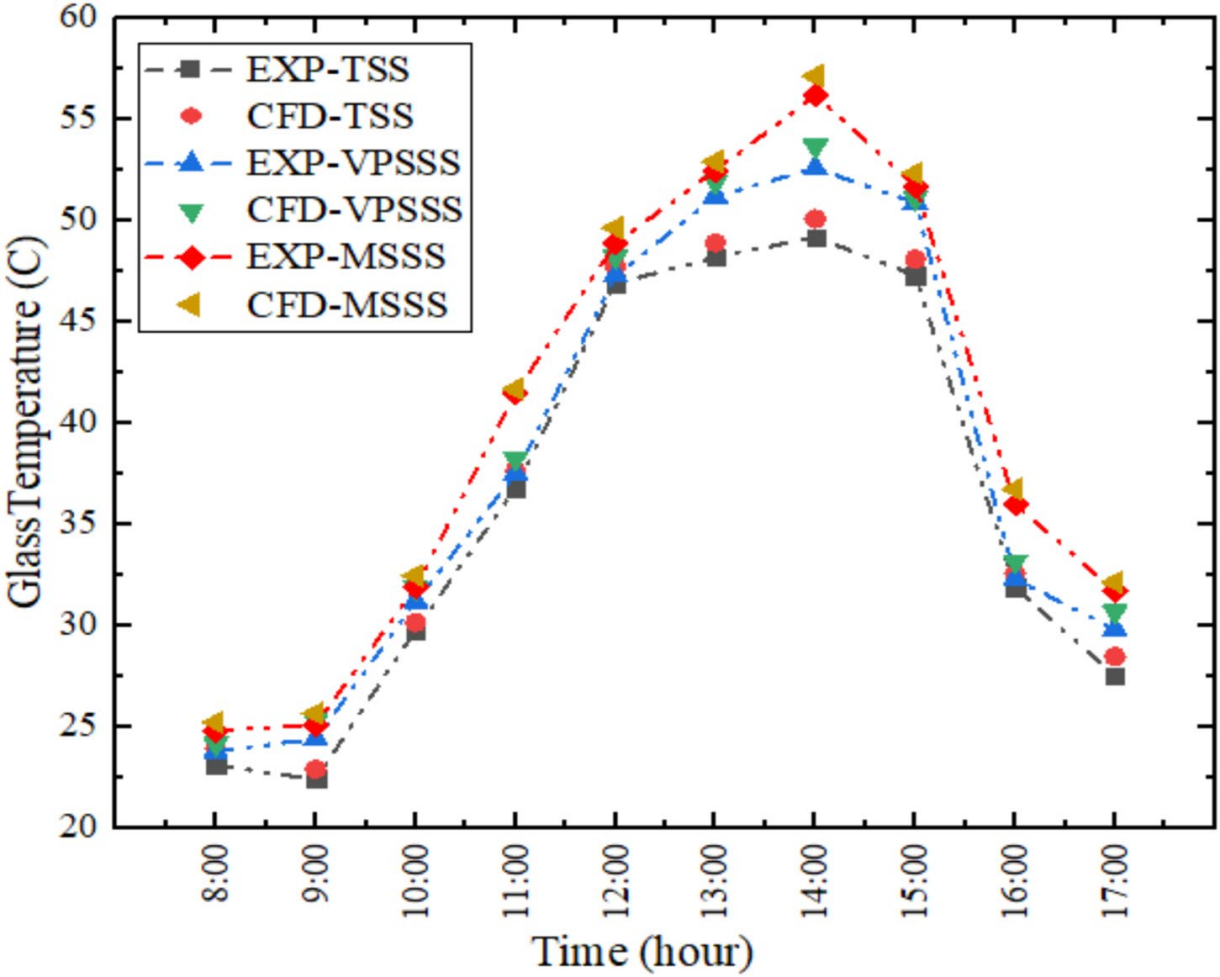
Glass temperature variation experimentally and numerically.

The changes in the glass temperatures experimentally and numerically calculated in the solar still are plotted in Fig. 7 where there is a close agreement between the two datasets (average relative error is approximately 2.36%). The temperature of the inner glass surface will always be below the temperature of the water and vapor because of the convection and radiations that will lose its heat to the colder ambient air. The main source of the driving force of the condensation process is this temperature difference between the warmer surface of water (around 57.863.1 °C) and the colder surface of the glass (around 45–55 °C), with water vapor condensing on the inner surface of the glass. The highest temperature of the glass is reached at about 2:00 PM, as per the patterns of the solar irradiance and in the melamine sponge-enhanced still (MSSS) due to the increased thermal absorption and evaporation rates leading to slight rise in the internal vapor and glass temperatures, as compared to the conventional still.

**Fig. 8:**
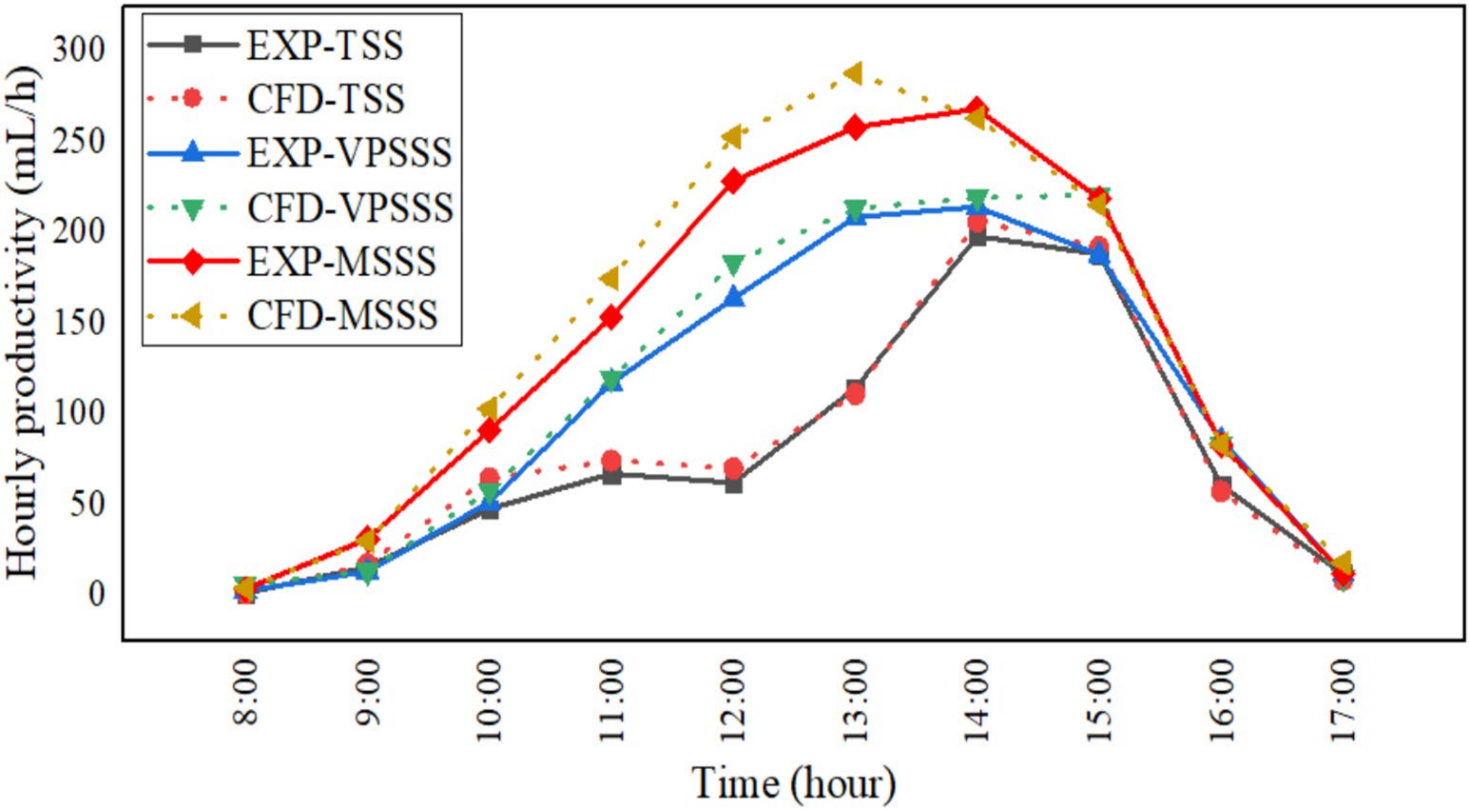
Hourly productivity experimentally and numerically.

The productivity of the three solar still configurations namely the traditional, pumice and melamine sponge enhanced (TSS, VPSSS, and MSSS respectively) are shown in Fig. 8, which illustrates the peak productivity in all three setups at approximate time of 2.00 pm. The highest productivity was attained by the MSSS (around 1347 ml/day), VPSSS (1055 ml/day), and TSS (858.7 ml/day). This improvement is mainly attributed to the fact that the porous nature of the material increases the surface temperatures of water: the high porosity (> 99 percent) and low thermal conductivity (~ 0.035 W/mK) of the melamine sponge maximizes the area of evaporation and minimizes the thermal loss, whereas the moderate porosity (~ 65%) and the high thermal mass (~ 500 kg/m^3^) of pumice stone enhance the heat retention and even distribution of capillary water. The similarity between experimental and numerical results (the error is approximately 2.36%) is the reason why Dunkle model should be applied since it proves the fact that the larger the difference in temperature between water and glass, the faster will be the rate of evaporation and condensation.

**Fig. 9:**
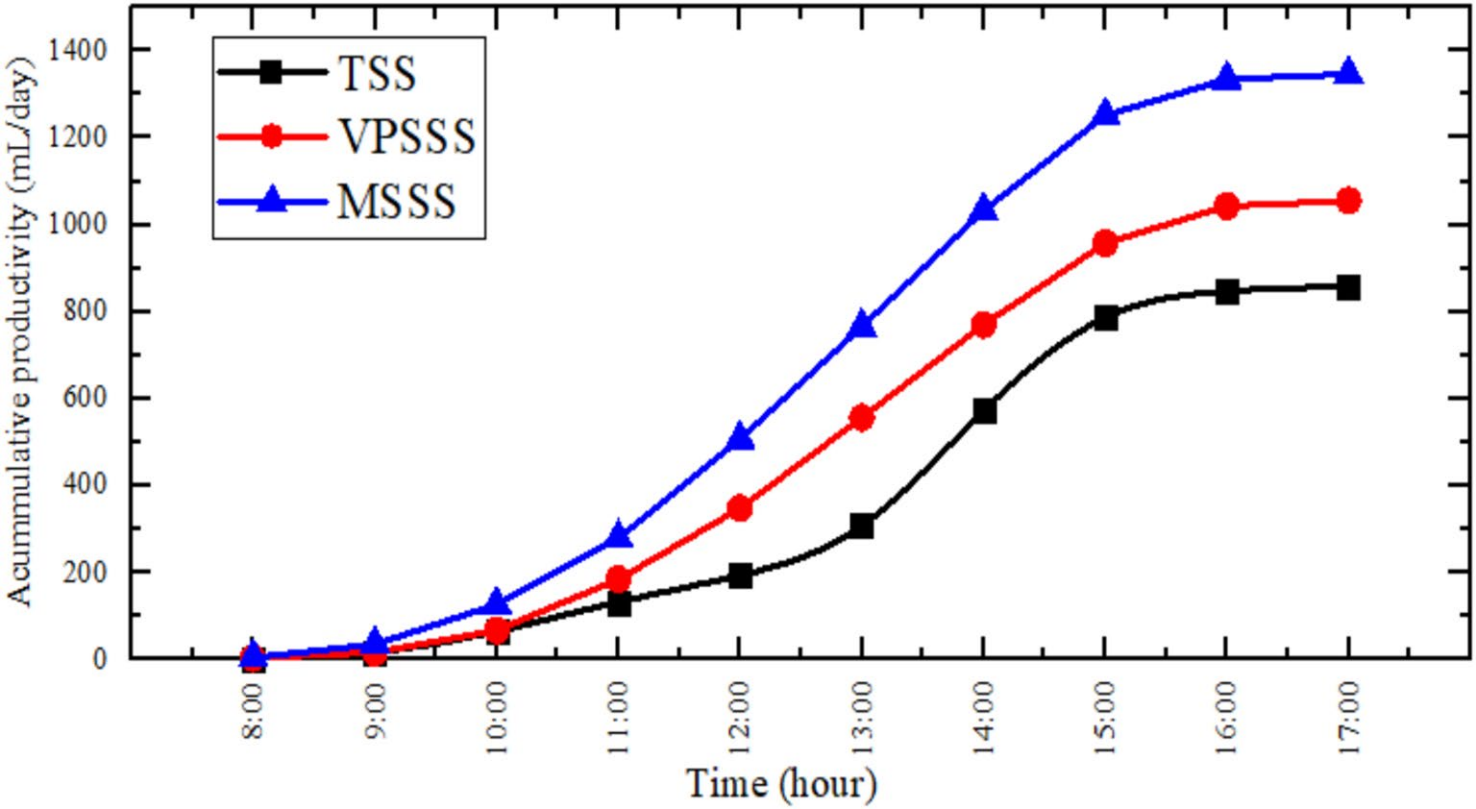
Accumulative productivity experimentally.

As Fig. 9 indicates, accumulative productivity of the three solar stills presents a higher number of products per day in MSSS (melamine sponge) (1347 mL), VPSSS (pumice stone) (1055 mL), and lastly the traditional still (TSS) (858.7 mL). This is because the porous characteristics of the physical materials allow them to be ranked in this order: the incredibly high porosity (> 99%) and low density (~ 8 kg/m^3^) of the melamine sponge maximize the evaporative surface area and evaporate water very quickly, whereas the moderate porosity (~ 65%) and increased density (~ 500 kg/m^3^) of the pumice stone allow it to retain a lot of heat and distribute water to the capillaries, which makes it more productive. The traditional still that was not porous had the least productivity because its surface area of evaporation was small and the heat was not evenly distributed.

**Fig. 10:**
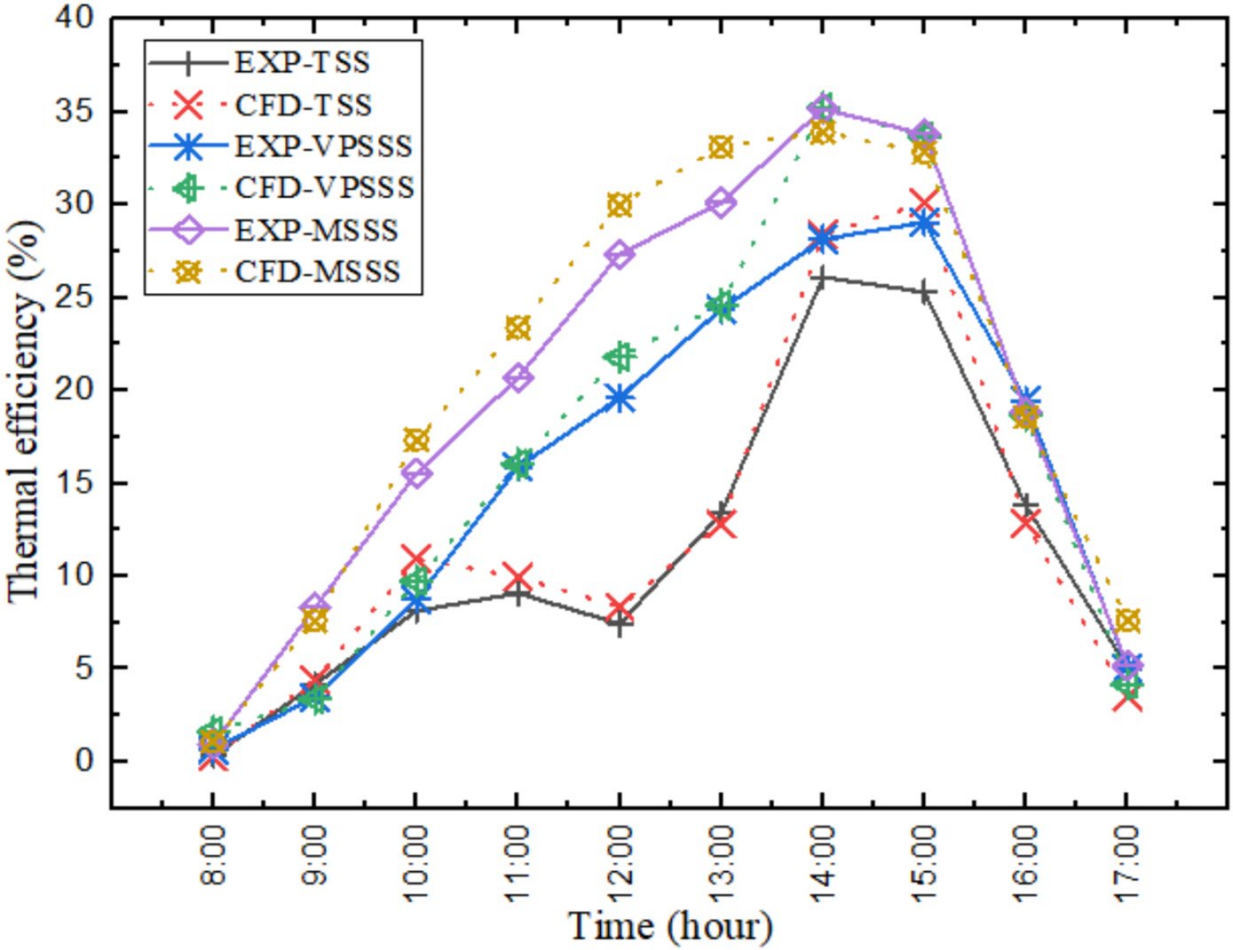
Hourly Thermal efficiency of solar still experimentally and numerically.

Figure 10 shows experimentally and numerically the hourly thermal efficiency of the solar still and indicates that the efficiency is at its highest near the maximum solar irradiance (e.g. about 45–50% in the MSSS still) at midday but falls in the early morning and later afternoon. The trend is also consistent with the profile of water temperature because when the water temperature is high (e.g. 63.1 °C in MSSS at 2:00 PM), the rate of evaporation increases because the difference in the vapor pressures between the water surface and the cooler glass cover increases. The porous materials and particularly melamine sponge further enhance efficiency by increasing the area of evaporation and increasing heat uptake to ensure better use of solar energy during maximum radiation periods.

**Fig. 11:**
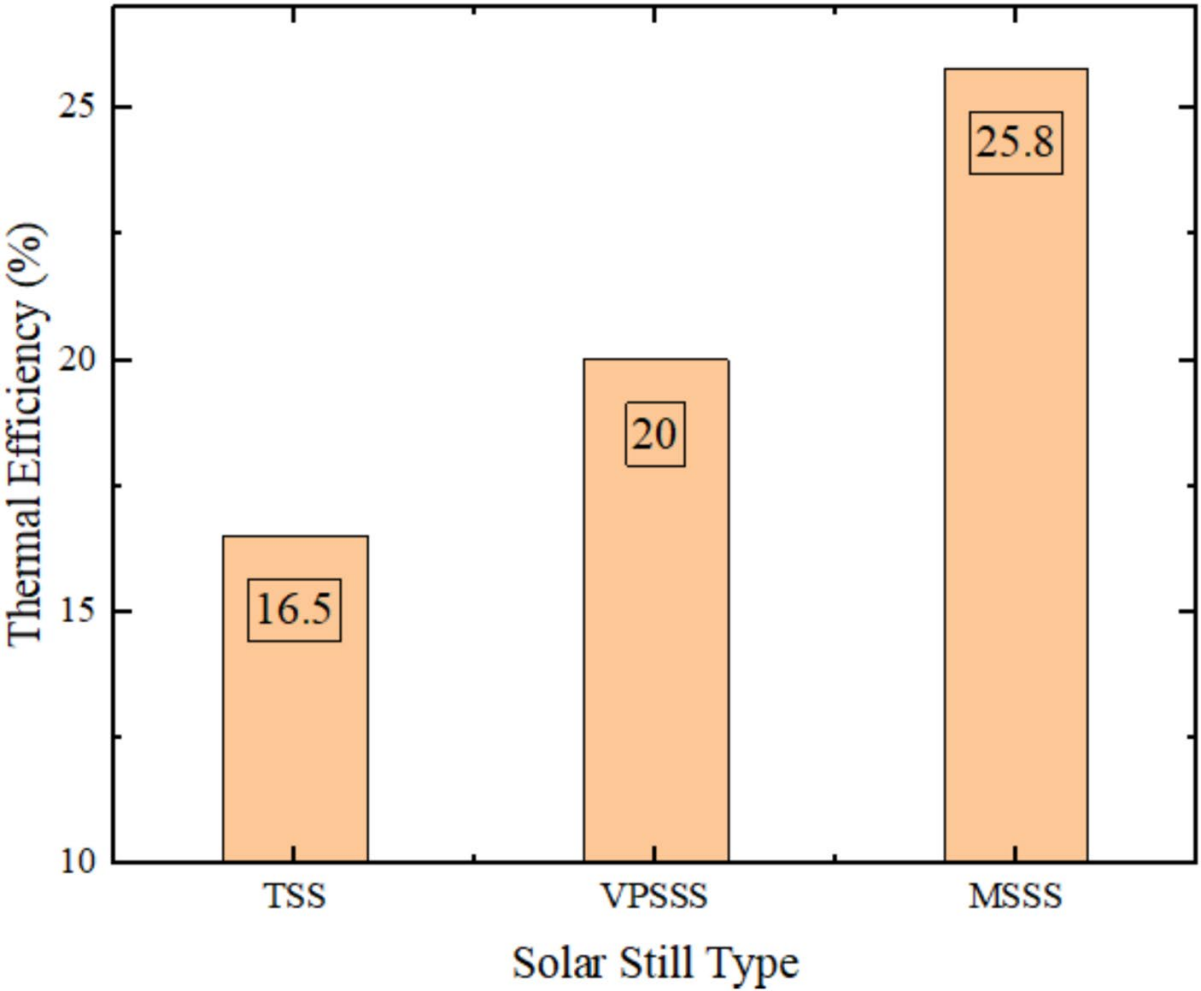
Average thermal efficiency experimentally.

Figure 11 shows the experimental values of average thermal efficiency of three solar still designs Traditional Solar Still (TSS), Pumice Stone-enhanced Solar Still (VPSSS), and Melamine Sponge-enhanced Solar Still (MSSS). The findings indicate that the TSS had an average thermal efficiency of about 31.5%, and the VPSSS and MSSS had thermal efficiencies of 38.2 and 49.3%, respectively, which were 21.2 and 56.4% of the baseline respectively. These improvements have been ascribed to the unique thermophysical characteristics of the porous materials: the extremely high porosity (> 99%), and the low thermal conductivity (~ 0.035 W/mK) of the melamine sponge encourage a higher evaporation surface area and faster evaporation, whereas the moderate porosity (~ 65%) and higher thermal mass of the pumice stone encourage heat retention and water distribution to the capillaries, respectively, which drive up water temperatures and, consequently, thermal efficiency. The ultra-high porosity (> 99%) and low thermal conductivity (~ 0.035 W/mK) of the melamine sponge to a considerable extent contribute to the high evaporation area per unit mass, and minimizes thermal losses, which directly contributes to the 17.8%-point improvement in efficiency over the TSS.

**Fig. 12:**
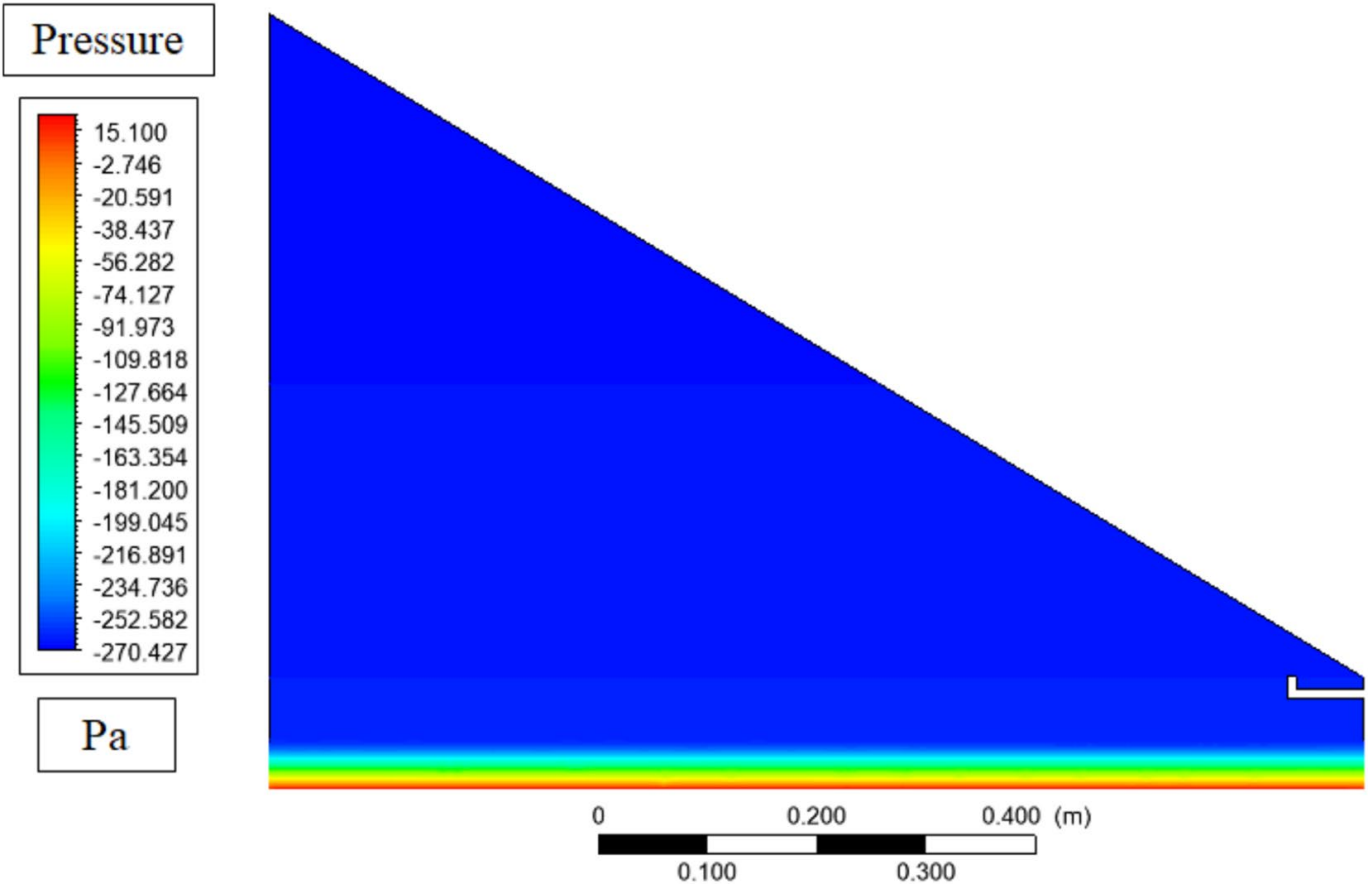
Pressure contours in solar still.

The pressure gradient that exists in the hydrostatics at the bottom of the basin is very evident in the pressure contours in the solar still as illustrated in Fig. 12, where the pressure reduces upwards to the glass cover. This is because this pressure difference, which is caused by the density variations due to temperature, is the main force behind vapor movement: at warmer water levels the local air density is smaller, as a result of higher temperature, which causes an upward convective movement, carrying vapor upwards to the colder condensation level. The contours indicate that the minimum pressures are around the glass where the condensation process occurs and thus sustain evaporation–condensation cycles which are crucial in the distillation process.

**Fig. 13:**
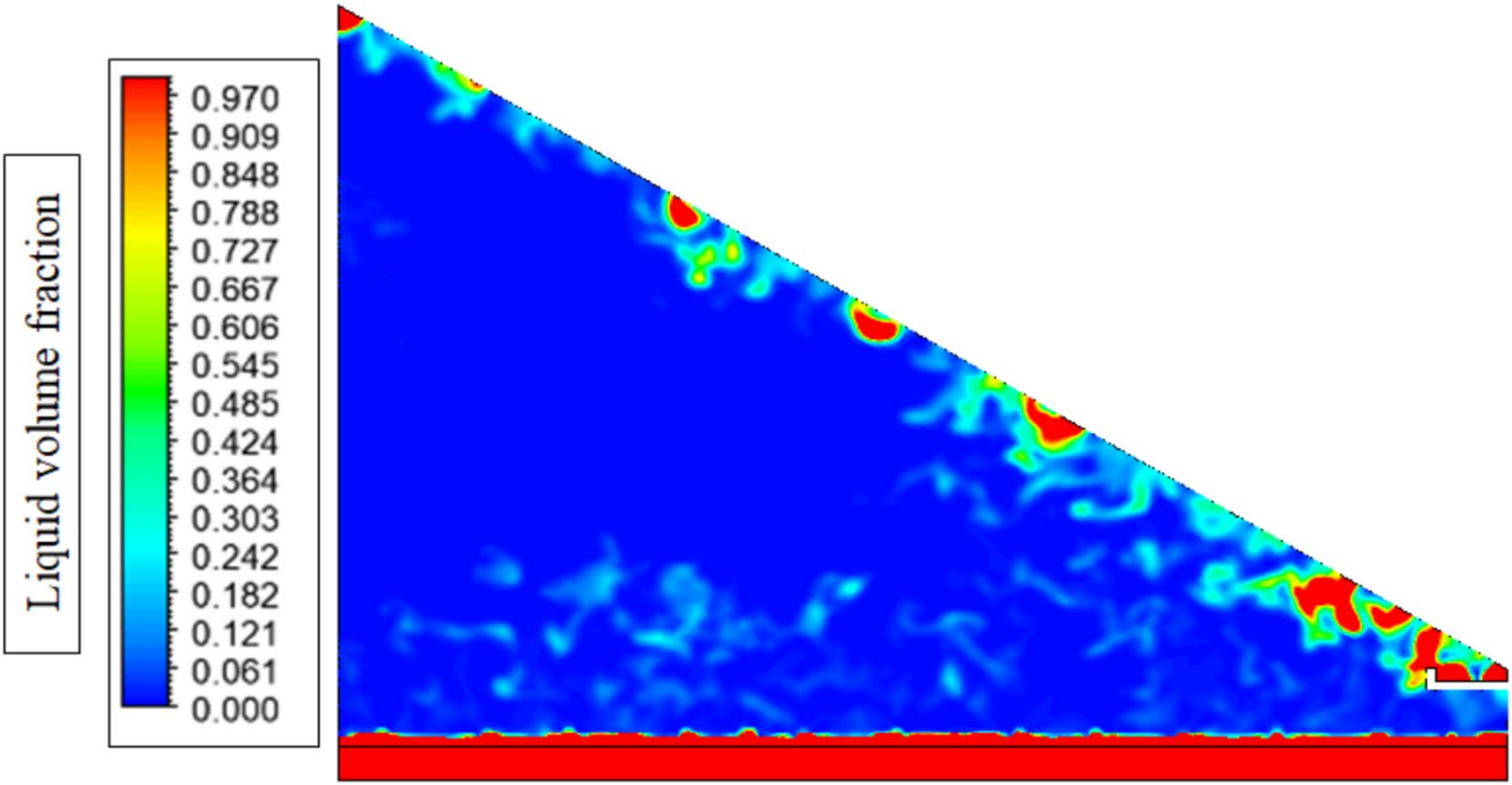
Volume fraction of water liquid contours in solar still.

Figure 13 depicts the contours of the volume fraction of the liquid water inside the solar still highlighting the evaporation process that takes place in the water layer at the free surface. Its contours depict that the liquid water (red/orange areas) is more concentrated at the bottom of the basin and becoming less concentrated, approaching the water–air interface (blue/green areas), where the evaporation is the most active. This gradient occurs due to the absorption of solar radiation, which causes the heating of the water, lift of the vapor pressure at the surface and evaporation. This vapor spreads out into the air above, and as far as the part of the cover reaches the colder glass, it condenses into drops, and these drop down to be collected. The clear separation of the liquid phase and the vapor phase can be visually confirmed and therefore the effective phase-change process that forms the basis of solar still functioning.

**Fig. 14:**
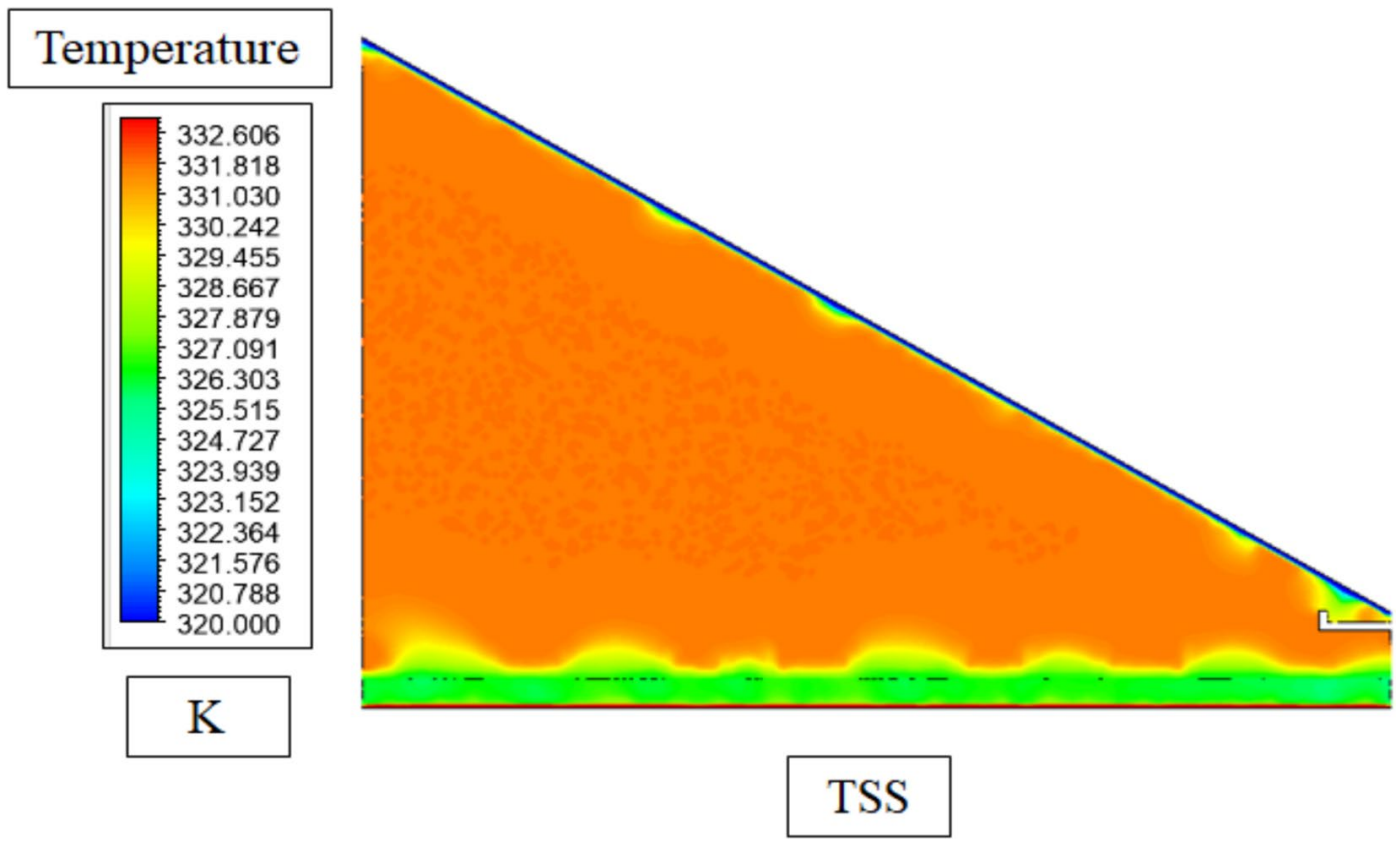
Temperature contours in TSS.

Figure 14 of the Traditional Solar Still (TSS) shows that the temperature contours are relatively narrow with three very distinct sections: the warmer region close to the absorber plate (intermediate temperatures), the hotter vapour in the centre because of latent heat loss during evaporation and the cooler region closest to the glass cover where the condensation process happens. Such a small thermal gradient (45–55 °C in the basin) is due to the lack of porous material that minimises the area of evaporation and heat capture. As a result, the thermodynamic force of evaporation temperature difference between water and glass is reduced and thus, reduced productivity as opposed to improved stills.

**Fig. 15:**
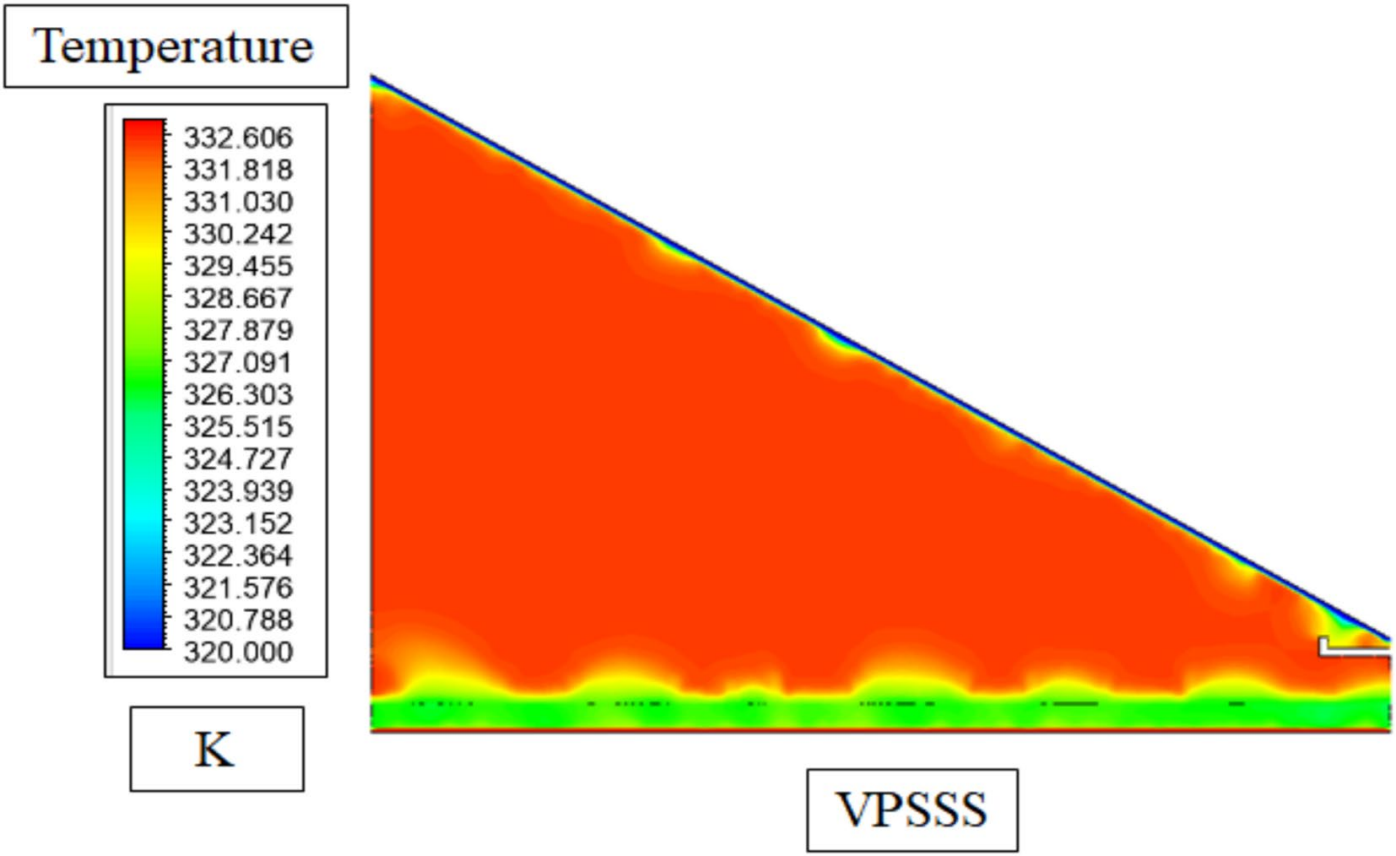
Temperature contours in VPSSS.

The temperature profile in the VPSSS (solar still with pumice stone) is represented in Fig. 15, indicating that there is a clear thermal stratification with maximum temperatures near the basin floor (around 58–62 °C) as a result of the direct solar absorption and the moderate thermal conductivity of pumice (around 0.15 W/mK) and its high density (around 500 kg/m^3^). The vapor zone in the middle is moderately hot (approximately 55–58 °C) due to the latent heat, which is released through the evaporation and the upper area close to the glass cover is colder (say 45–50 °C), and this forms a temperature gradient, which is causing the condensation. Its porous structure enhances capillary water flow, thermal inertia, providing better and more homogenous temperature field in the pumice stone than in the traditional still, which promotes sustained evaporation and increased productivity.

**Fig. 16:**
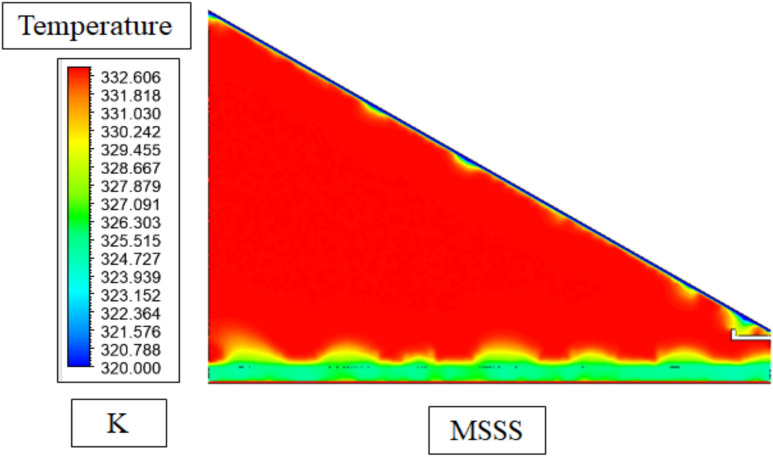
Temperature contours in MSSS.

The contours of temperatures in the Melamine Sponge Solar Still (MSSS) in Fig. 16 indicate a clear thermal stratification and three main parts including the hottest areas (up to about 63.1 °C) at the absorber plate and in the vapor zone, a middle range of temperatures in the water basin and the coldest area in the glass cover. The high thermal profile is physically explained by the high porosity (> 99%) and low thermal conductivity (~ 0.035 W/mK) of the melamine sponge which maximizes the surface area of the evaporative interface and facilitates efficient local heating of the air–water interface. Consequently, the sponge allows better heat retention and stronger evaporation than the traditional still, which has resulted in the higher temperatures and stronger thermal gradients that result in further condensation and greater productivity.

**Fig. 17:**
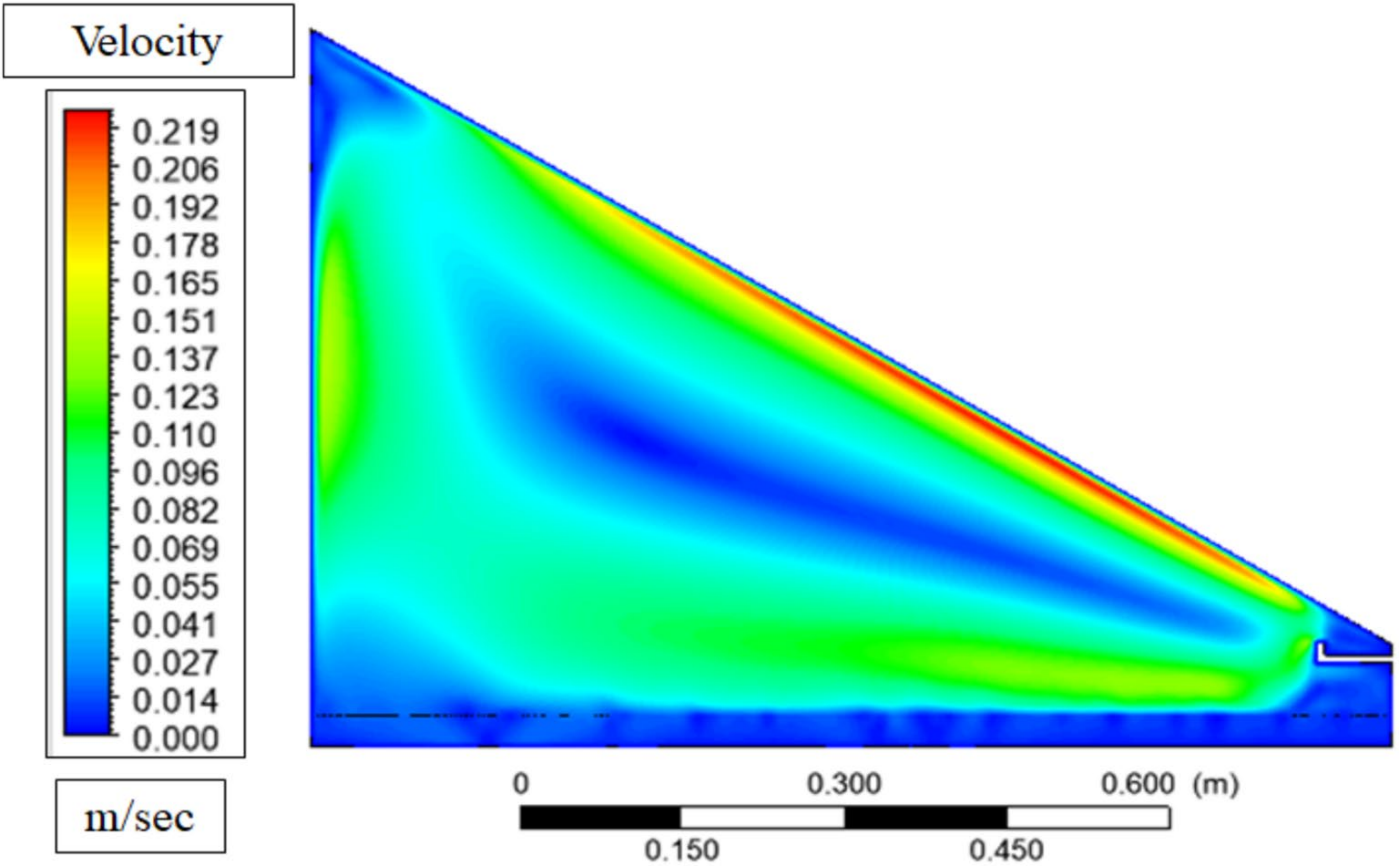
Velocity contour before evaporation.

Figure 17 shows the velocity contour of the solar still prior to the considerable evaporation taking place representing a natural circulation pattern of air. This movement is also caused by density gradients: the air that comes into contact with the cooler glass surface becomes heavier and sinks, and the warmer and lighter air that approaches the water surface ascends forming a convective circle. This circulation increases the movement of air over the water surface, compelling the system to eagerly undergo mass transfer when evaporation has started, but at this point, the circulation is largely laminar and no longer subject to turbulence due to the disruption of the release of vapor.

**Fig. 18:**
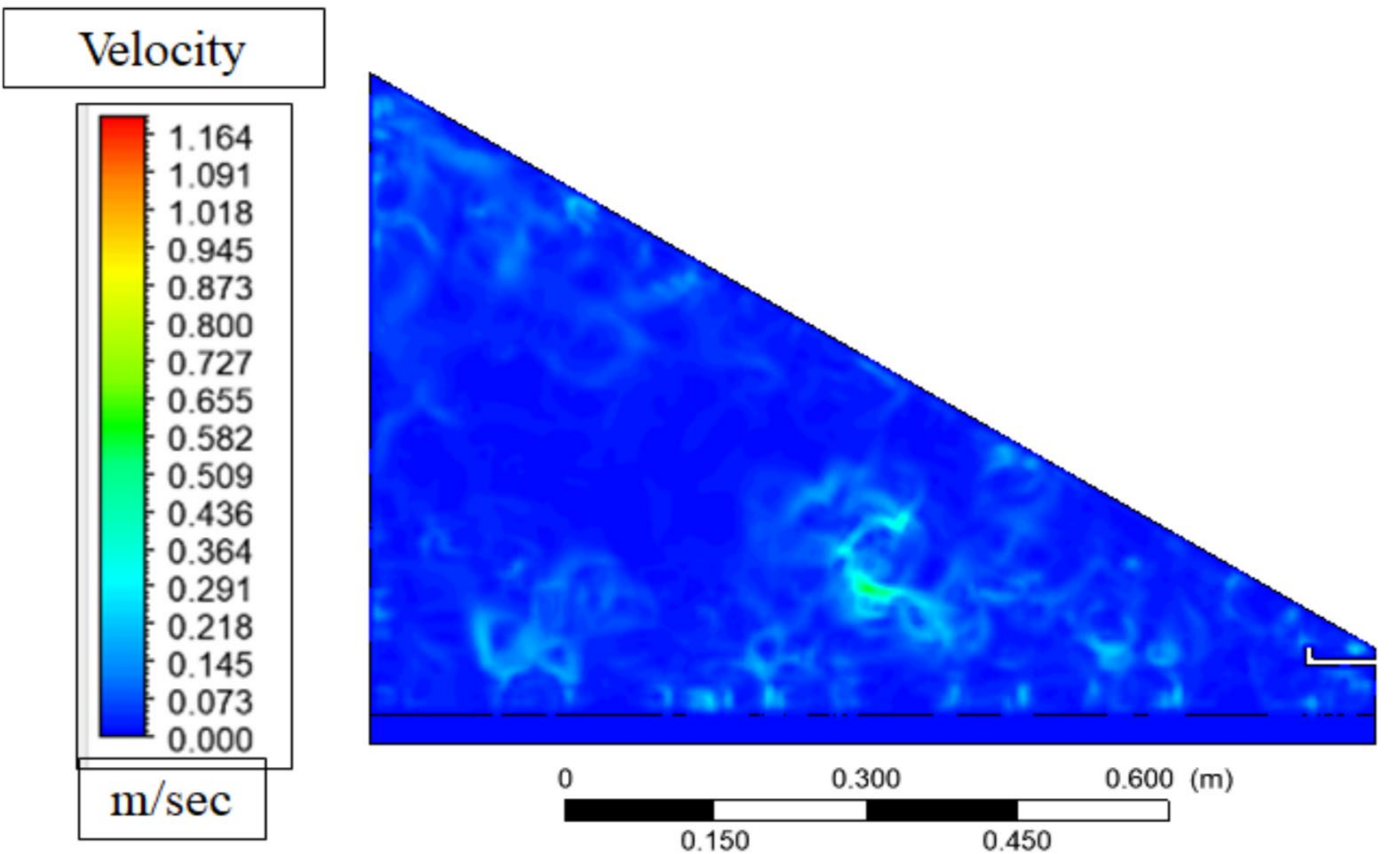
Velocity contour after evaporation.

Figure 18 of the velocity contour following evaporation indicates a more turbulent and energetic flow field than the flow field was in Fig. 18, with higher velocity magnitudes close to the water surface and the glass cover owing to the convection driven by buoyancy and vapor transport. This distortion is due to the warm and heavy moisture-filled vapour that ascends upwards in the heated water surface, forming an upward convective circulation that increases in velocity as it takes in latent heat through evaporation to a speed of a maximum of about 0.25 m/s at the surface of the glass. Its increased movement promotes the transfer of vapor to the cooler glass surface where it condenses and the downward movement of heavier, colder air along the glass finishes the circulation loop, which equates to an increased thermal and mass transfer efficiency of the still.

### Cost evaluation analysis

**Fig. 19:**
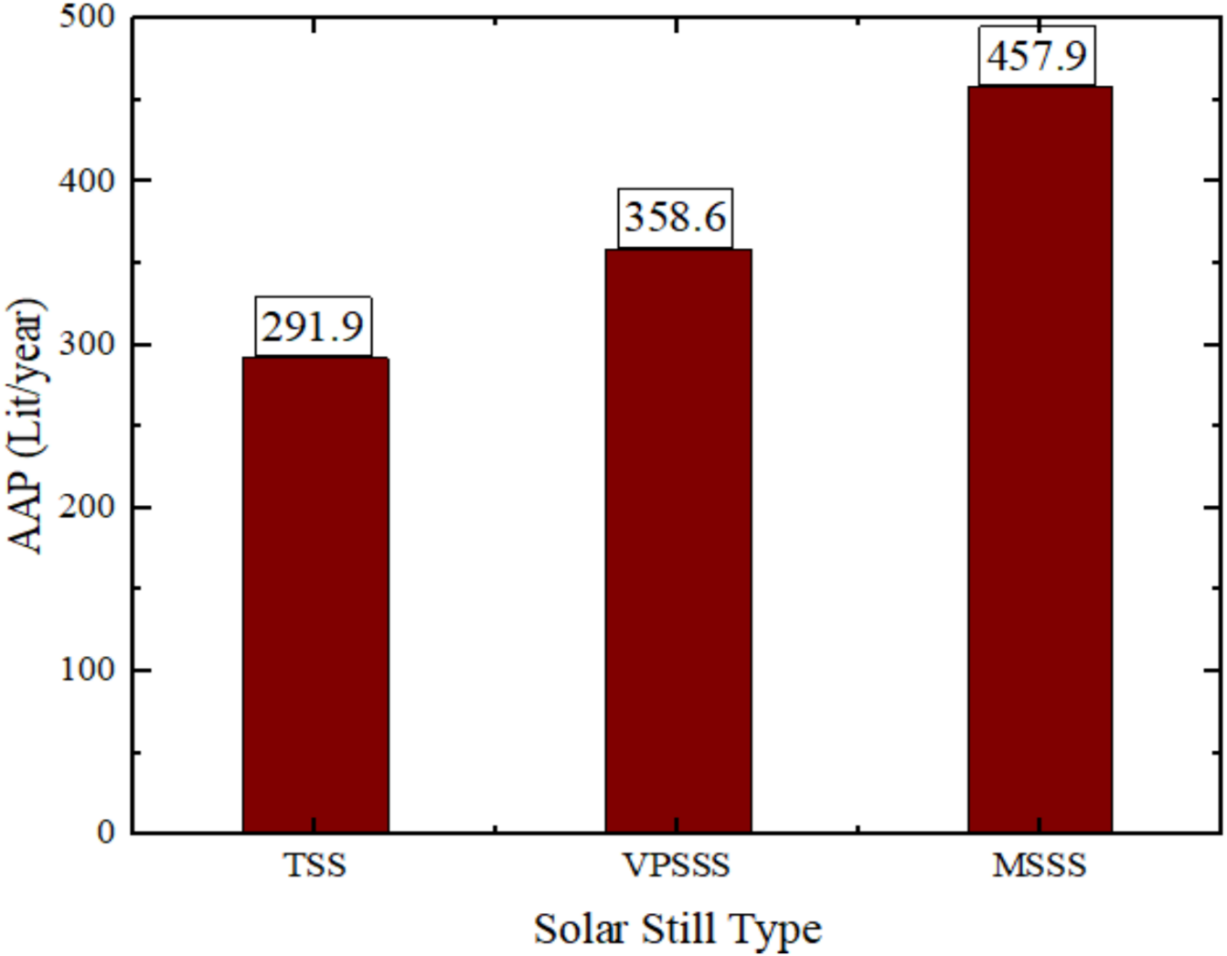
Annual production values for the three stills.

The three solar still designs i.e. the Traditional Solar Still (TSS) resulted in a production of 292.5 Liters/year, the Pumice Stone-enhanced still (VPSSS) had 358.7 Liters/year and the Melamine Sponge-enhanced still (MSSS) had 457.9 Liters/year fresh water production per year as shown in Fig. 19. This order of performance is explained by the specific thermophysical characteristics of the porous materials, i.e. the very high porosity (> 99) and the large air–water interface of the melamine sponge, which sharply increase the total area and efficiency of the evaporation surface producing the highest yield. The moderate porosity stone (pumice, medium) and increased thermal mass increase the sustainability of heat and capillary-driven water allocation and lead to a moderate productivity gain. The traditional, on the other hand, is not equipped with these improvements that would result in reduced evaporation and the least yearly output. The high yield of the MSSS (457.9 L/year) is due to its high porosity (> 99%), which gives the MSSS an optimal evaporative interface and capillary distribution which directly translates the increased solar absorbance into more distillate yield.

**Fig. 20:**
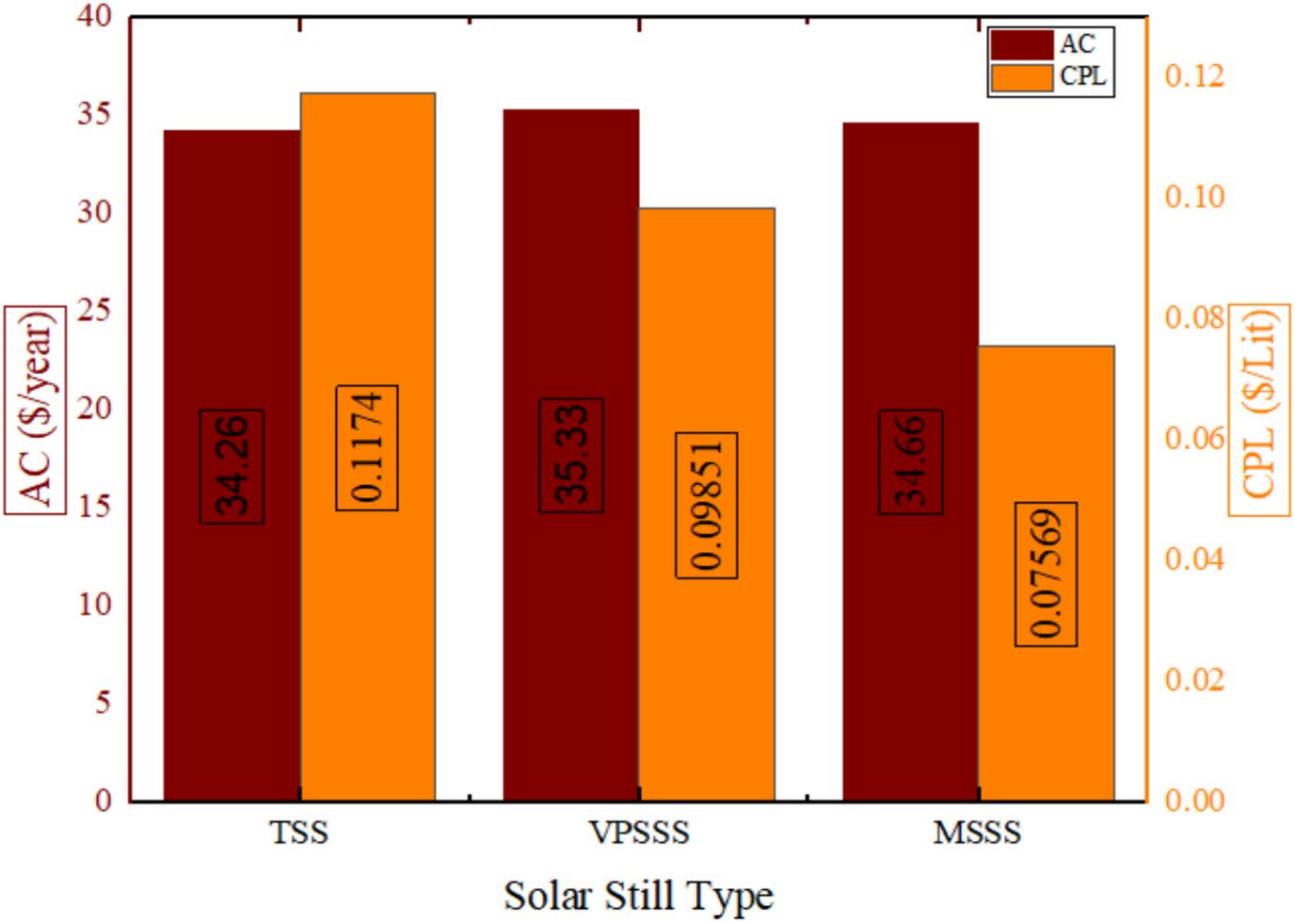
Annual cost and cost per Liter for three still.

Figure 20 depicts the annual costs and cost per Liter of the three solar still configurations and indicates that the MSSS (melamine sponge) system is the lowest at being 0.07569 $/L, which is a 35.5% reduction of the conventional TSS (0.1174 $/L), whereas the VPSSS (pumice stone) system is the lowest at 0.09851 $/L, which is a 16% reduction. This is mainly because of the huge rise in productivity, MSSS and VPSSS yielded 56.9% and 22.9% more distillate respectively without a corresponding rise in the capital expenditure. The increased porosity and thermal conductivity of the porous materials increase the rate of evaporation and thermal retention and therefore yields better output and dilutes the fixed annual costs (capital recovery, maintenance and salvage) per Liter. The reason of the low CPL of MSSS (0.07569 $/L) is due to its higher productivity per annum (457.9 L/year) which evenly shares the fixed annual costs even when it has a marginally greater initial capital investment.

### Payback period

**Fig. 21:**
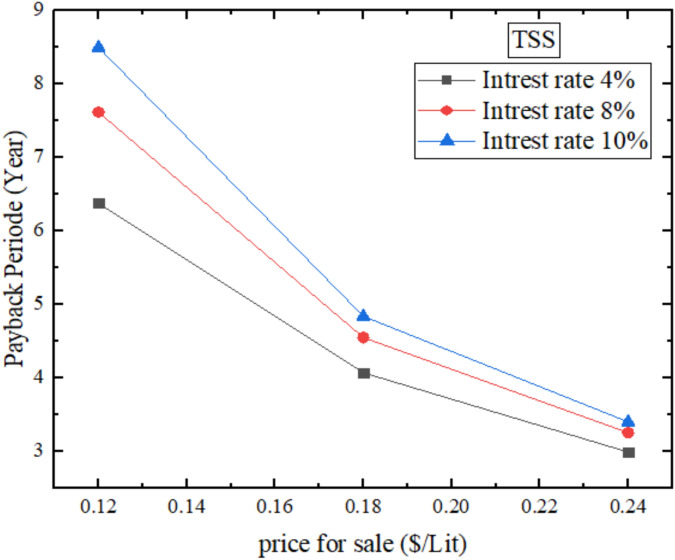
Annual financial payback period for TSS.

Figure 21 depicts annual monetary payback period of the Traditional Solar Still (TSS) at different interest rates (4, 8, and 10%) and prices of water at sale (0.12–0.24 $/Liter). The payback period will take about 4 years at an interest rate of 4% and a selling price of 0.18 $/Liter. The comparison of this quite moderate payback period to the initial cost of the TSS ($196.2) and its relatively low daily productivity (858.7 mL/day) is explained by the fact that the TSS has only a lower initial cost but a reduced annual productivity as well. The physical reason behind such performance is lack of improvement materials and as such, the water temperatures (maximum of about 57.8 °C) and the rate of evaporation is lower than modified stills. The payback period increases as interest rates rise or selling prices fall and as a result, it is clear that the conventional solar stills are economically sensitive to the market and financial situations, as the cost of financing and the cash flows per year are expected to be low. The lower interest rates have also contributed to the shorter payback period by offering lower financing costs that enhance the net cash flow per year compared to the initial investment of the capital investment of 196.2$that boosts cost recovery.

**Fig. 22:**
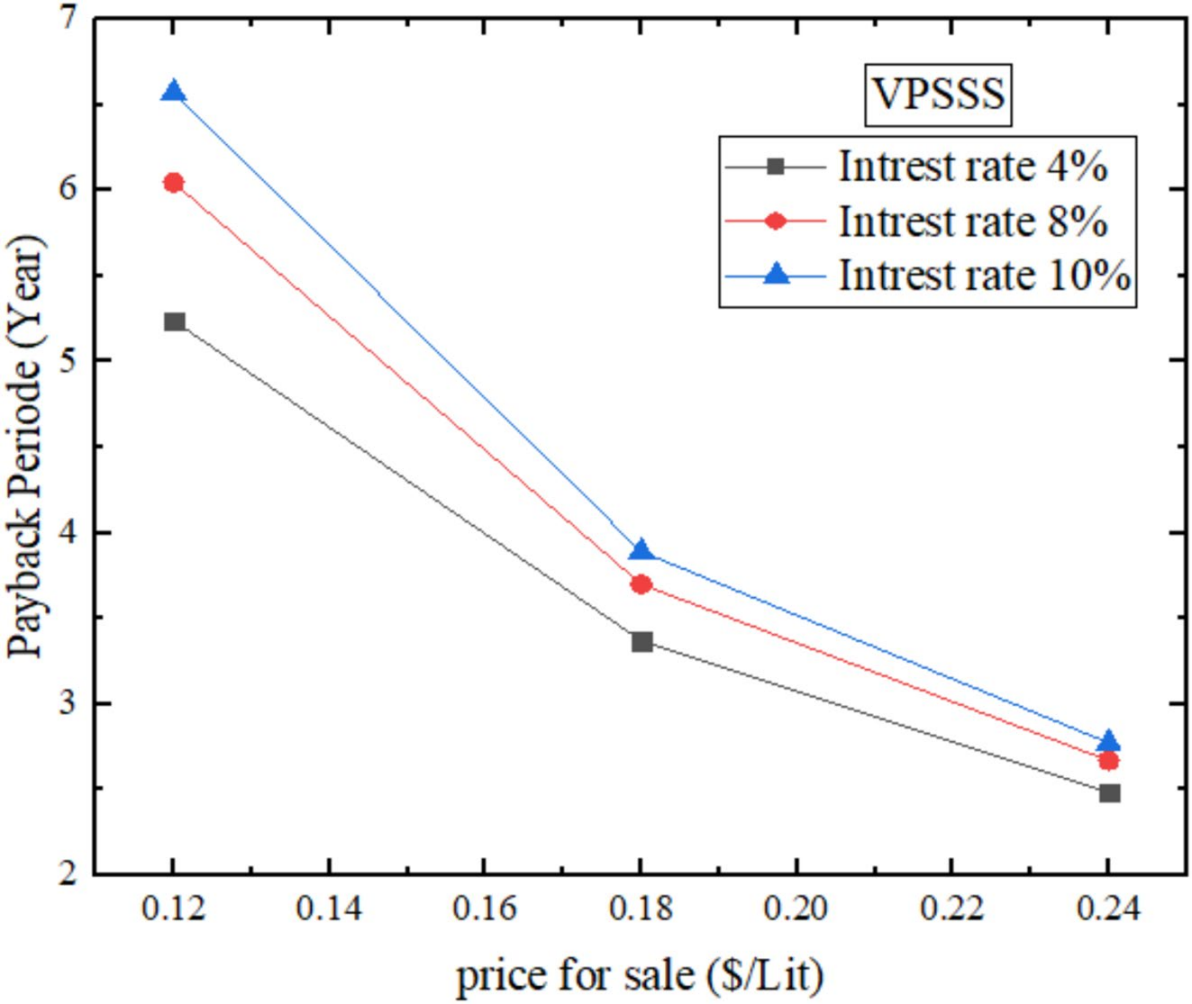
Annual financial payback period for VPSSS.

According to Fig. 22, the annual financial payback period is VPSSS (solar still enhanced with pumice stone), therefore with an interest rate of 4% and a selling price of 0.18 $/Liter, the payback period is 3.36 years as opposed to 4 years of the conventional still. This is attributed to the fact that the VPSSS has higher daily productivity (1055 mL/day) than the traditional still (858.7 mL/day) because pumice stone has moderate thermal conductivity (~ 0.15 W/mK) and porosity (~ 65%), which improves thermal retention and water distribution by capillary, which in turn increases evaporation and freshwater yield. This increase in production of the production per annum makes the cost less per Liter and brings about the recession of the initial cost quicker. The reason behind the lower payback period (3.36 years) is that pumice stone is characterized by medium-porosity (approximately 65%) and thermal conductivity (approximately 0.15 W/mK), increasing the distribution of capillary water and thermal retention, which increases the annual productivity and cost recovery in a shorter time.

**Fig. 23:**
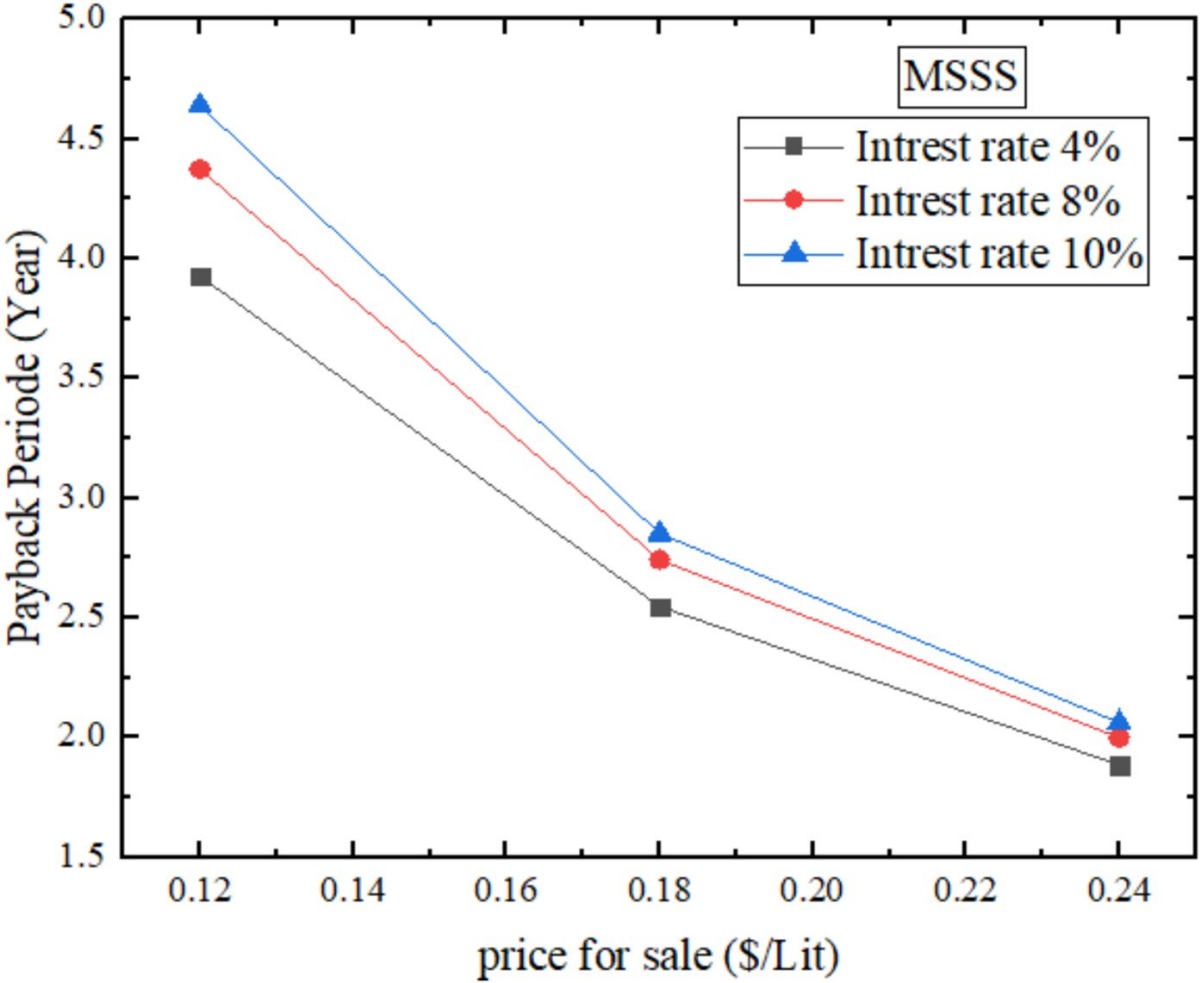
Annual financial payback period for MSSS.

Figure 23 demonstrates payback period of the MSSS (melamine sponge-enhanced solar still) in terms of annual finances, with interest rate of less than 4% and selling price of less than 0.18$/L, the payback period is about 2.5 years. It is also driven by the fact that the MSSS shortens the payback period because the melamine sponge has a very high porosity (> 99%), and low thermal conductivity (~ 0.035 W/mK) that facilitates evaporation through maximizing the air–water interface and promoting thermal distribution. This leads to more rapid growth of revenue which is facilitated by the high freshwater yield, enabling the initial investment expenditure of $198.5 to be recouped sooner than in traditional systems or pumice enhanced systems. The ultra-high porosity (> 99%) and low thermal conductivity (= 0.035 W/mK) of the melamine sponge maximizes the evaporation, maximizing productivity (457.9 L/yr) with ideal financial circumstances, shortening the payback period to 2.5 years.

### Energy matrices

**Fig. 24:**
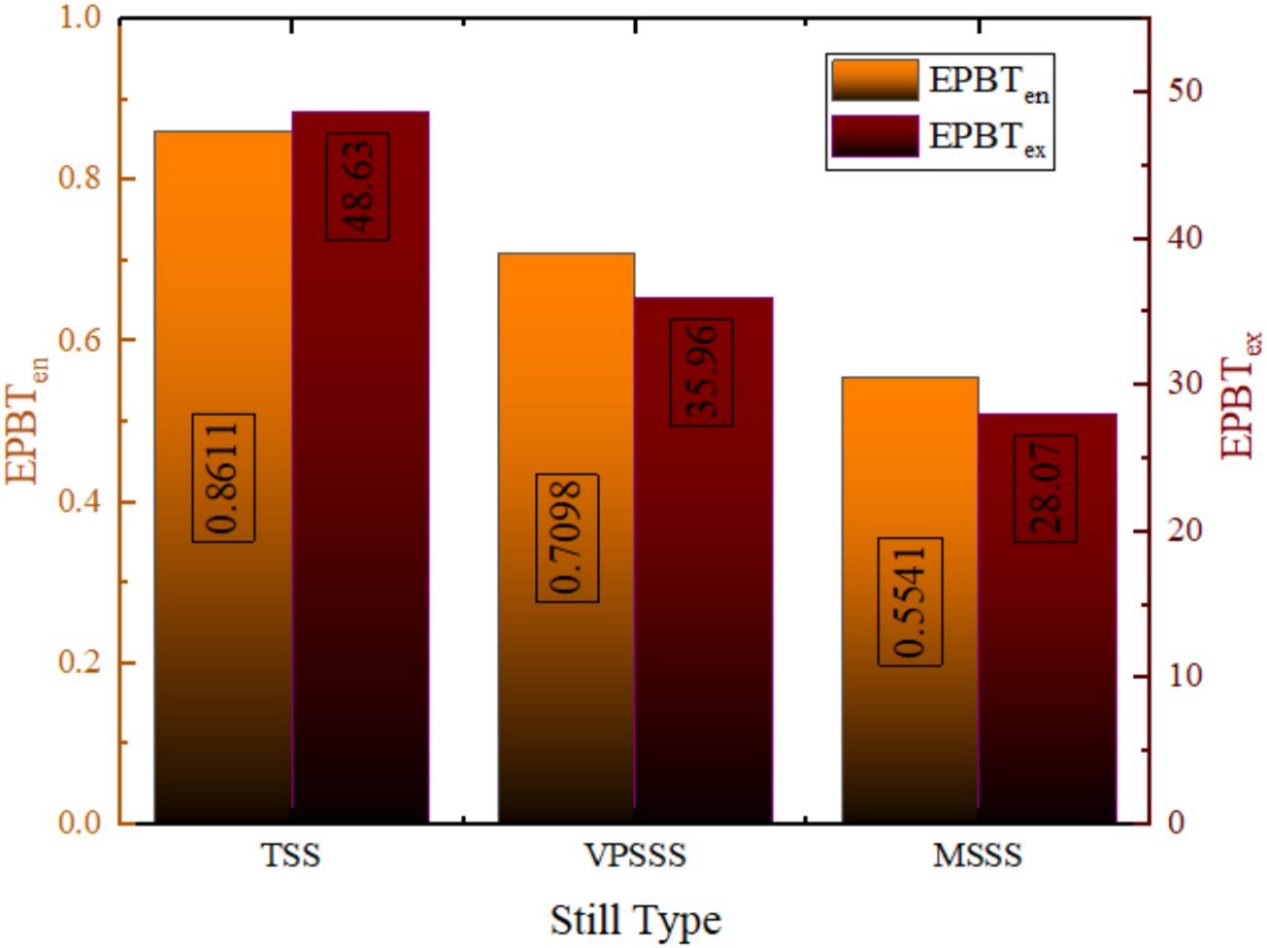
EPBT values ​​on an energy basis and on an exergy basis.

As illustrated in Fig. 24, the Energy Payback Time (EPBT) is much lower on an energy basis rather than exergy basis across the entire solar still configurations (TSS: almost 0.86 years energy vs. over 10 years exergy; VPSSS: almost 0.7 years energy vs. almost 35 years exergy; MSSS: almost 0.55 years energy vs. almost 28 years exergy). This difference is explained by the fact that energy analysis does not take into account the quality and utility of the energy generated, as the amount of thermal energy generated is relatively large, thanks to latent heat of evaporation, but the exergy analysis does take it into account, as the quality of energy generated by solar distillation at low temperatures is low, and extra losses that occur during the process are irreversible. The modified stills (VPSSS and MSSS) have lower values of EPBT in the two bases because they have improved productivity, and this elevates useful energy/exergy production compared to the energy/exergy used in their material. The high difference between the energy and exergy EPBT is due to the low grade thermal energy of solar stills which consistent useful work potential (exergy) is limited by high thermodynamic irreversibility as compared to total heat (energy).

**Fig. 25:**
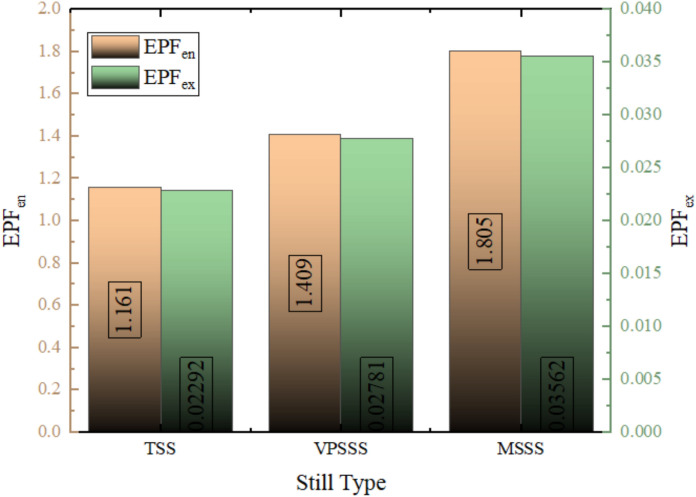
EPF values ​​on an energy basis and on an exergy basis.

The values of the Energy Production Factor (EPF) represented in Fig. 25 are relatively greater on energy basis scale compared to exergy basis scale as they demonstrate the inherent distinction between the quantity of energy and quality of energy. In case of the standard solar still (TSS), the EPF is close to being 1.16 on an energy basis, i.e. producing 16% more energy than the construction embodied, and on an exergy basis it is only around 0.2, implying that 20% of the embodied exergy is recaptured each year. This is the difference between energy analysis and exergy analysis: in the former, the total thermal energy is taken into account (by latent heat of evaporation), whereas useful work potential is taken into account in the latter (thus, the thermodynamic irreversibility and the low-grade quality of solar thermal distillation limit the useful work potential). The modified stills (VPSSS and MSS) are characterized by increased values of EPF because of the better thermal performance of porous materials, although exergy-based EPF is lower, which highlights the difficulty of tapping low-temperature heat into usable useful work. The high ratio of EPFenergy to EPFexergy (e.g., 1.16 vs. 0.20 of TSS) is due to the fact that exergy takes into consideration the low grade thermal energy low work potential, which is one of the major thermodynamic limitations of solar distillation.

**Fig. 26:**
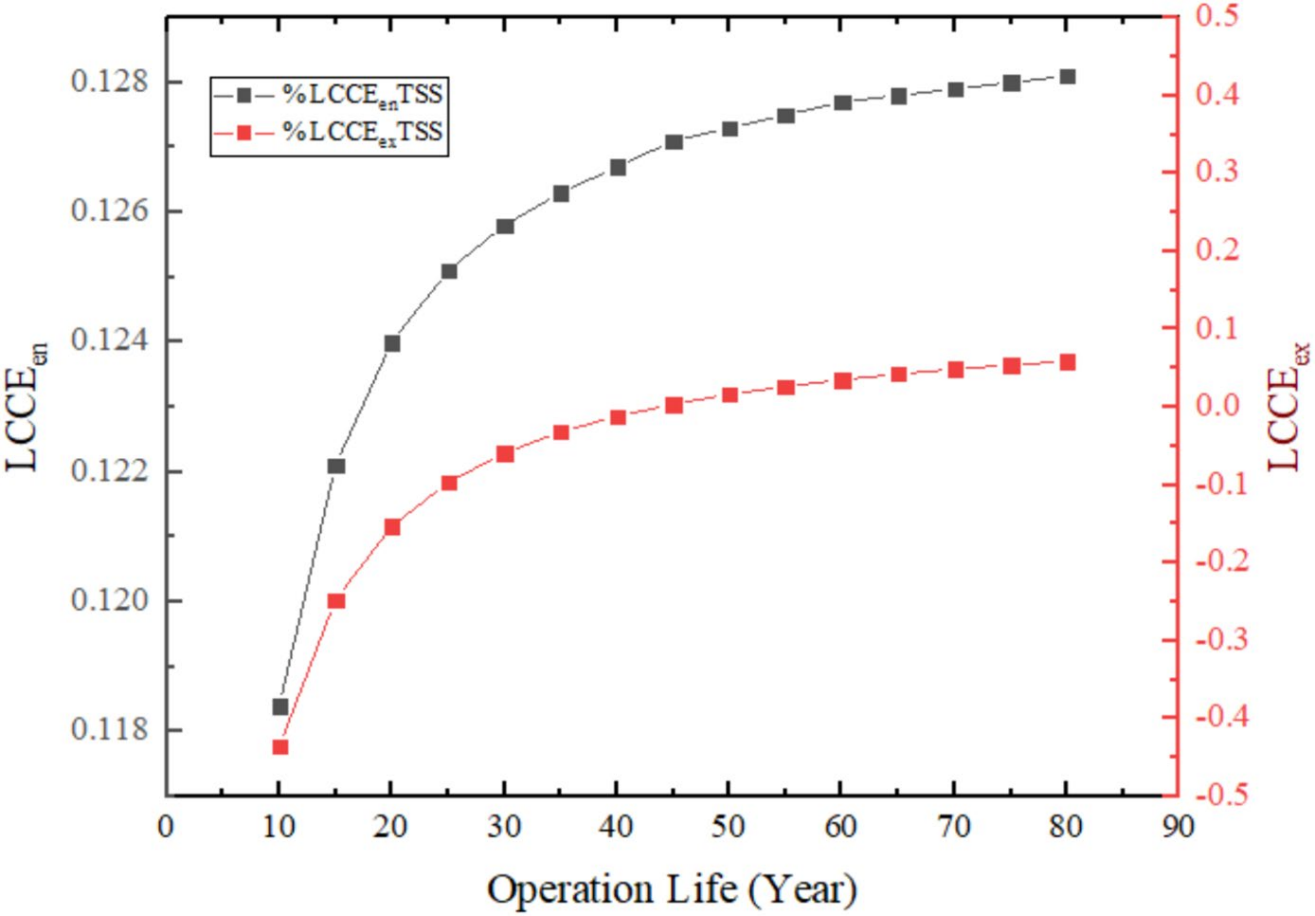
LCCE diagrams calculated on an energy and exergy basis over the system’s lifetime for TSS.

Figure 26 depicts the Life Cycle Conversion Efficiency (LCCE) of the Traditional Solar Still (TSS) in 10 years of operation with an energy-based LCCE of about 11.84% and negative exergy-based LCCE. The positive energy LCCE shows that the system is utilizing approximately 11.84% of the incident solar energy into useful thermal energy to evaporation, which indicates that the system is capable of recovering the embodied energy within the materials used in the system during the time of operations. Conversely, the negative exergy LCCE is due to the qualitatively low quality of thermal energy utilized in the process of distillation, with exergy being the potential useful work of the system; likewise, the low-grade heat produced by the system (approaching ambient temperatures) results in low exergy efficiency, and thus, after 10 years the TSS cannot recover all of the exergy contained in its materials, and a positive exergy balance is reached only after an estimated 48 years. This difference points to the thermodynamic constraint of the solar stills, the amount of energy is effectively used, yet the energy quality is low enough to support high exergy recovery. The negative exergy LCCE in 10 years point to the fact that even when there is positive energy recovery, the low grade of thermal energy cannot make up the high quality exergy that is contained in the manufacturing process in this period of 10 years.

**Fig. 27:**
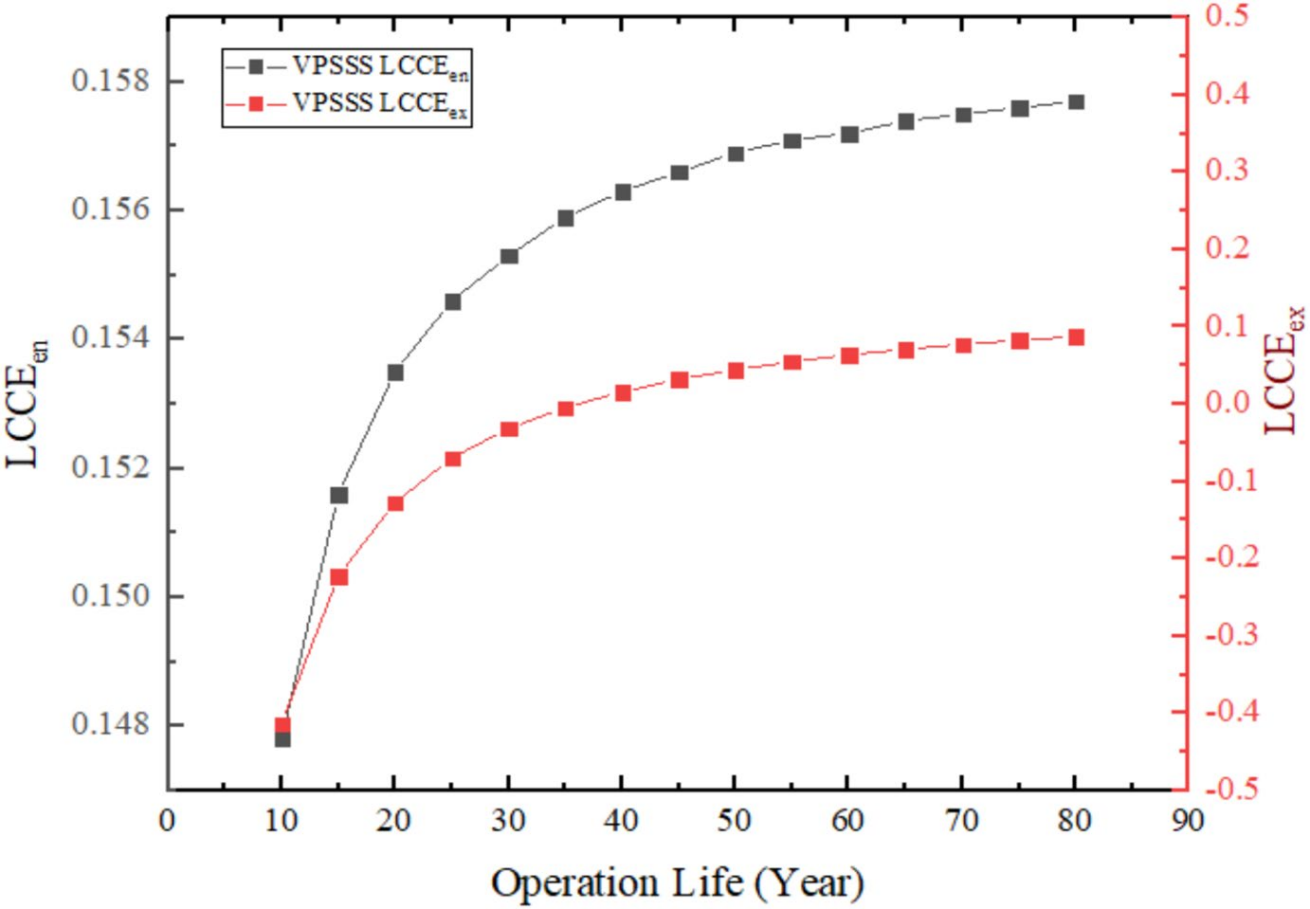
LCCE diagrams calculated on an energy and exergy basis over the system’s lifetime for VPSSS.

Figure 27 shows the VPSSS system (solar still with pumice stone) Life Cycle Conversion Efficiency (LCCE), indicating that exergy-based LCCE values are negative and less at the initial years compared to energy-based LCCE that has positive values. This is because the embodied exergy (quality energy applied in the manufacturing) surpasses the useful exergy generated each year, where low grade thermal energy is used in the distillation process. With time, when cumulative exergy output grows, the exergy based LCCE reaches a positive point with a recovery period that takes up to 35 years. Conversely, energy-based LCCE is positive across the board to a high of 14.78% at 10 years because the system is able to convert incident solar energy into usable thermal energy even though of low quality.

**Fig. 28:**
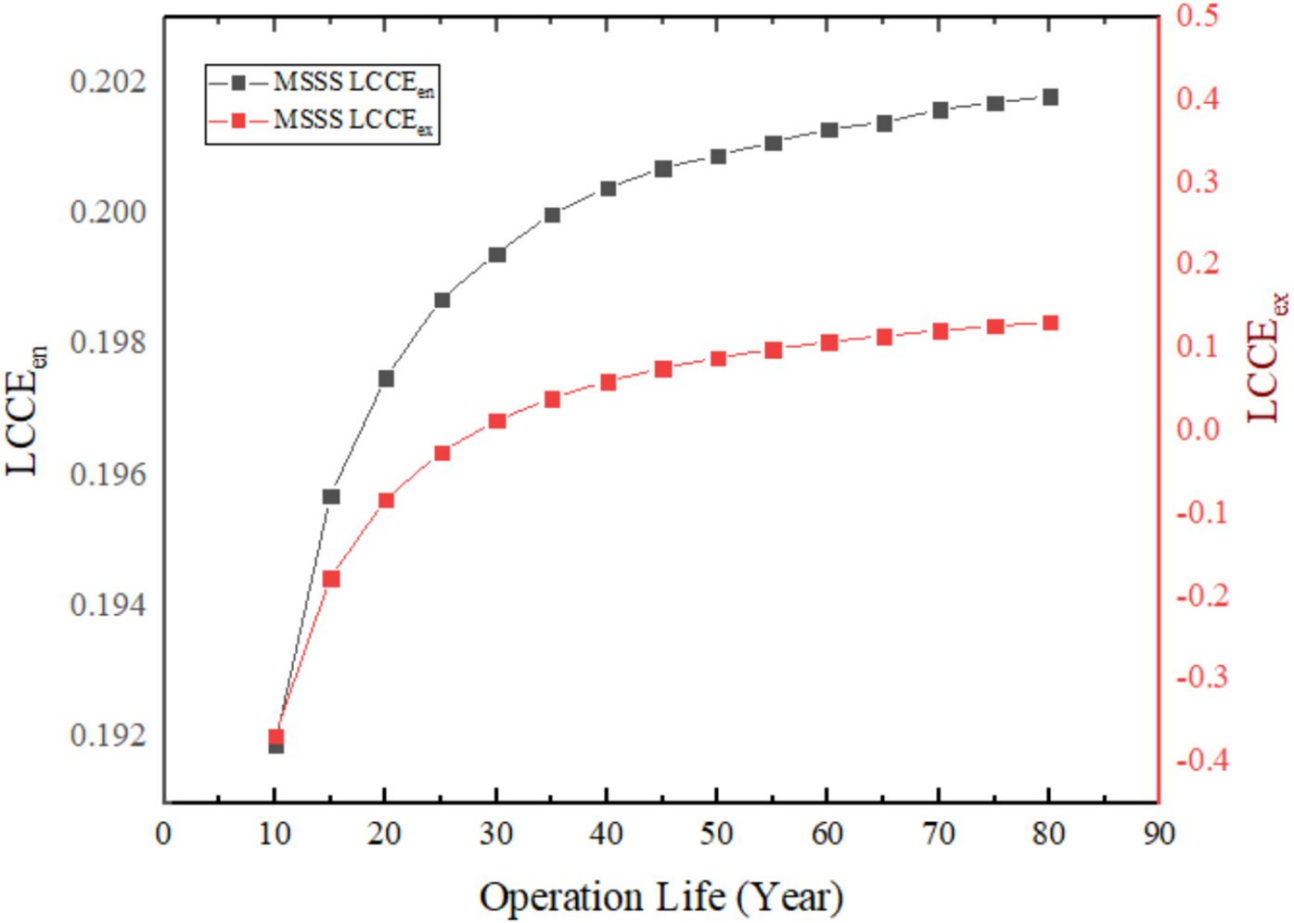
LCCE diagrams calculated on an energy and exergy basis over the system’s lifetime for MSSS.

The life cycle conversion efficiency of the melamine sponge solar still ( MSSS) over a 10-year period as presented in Fig. 28 demonstrates that the sponge high porosity (> 99) and low thermal conductivity (~ 0.035 W/m K) yield high evaporation rates and thermal retention that increases energy-based LCCE to 19.19%, which is a 62% improvement over the normal solar still. Nevertheless, the exergy-based LCCE is negative in this time, and the recovery time is estimated to be around 28 years, which is the debased quality of the thermal energy applied in the distillation process and the high embodied exergy in the system materials, despite the high productivity. Such a difference implies the trade-off between the higher yield of water and exergy efficiency, which needs additional optimization to enhance the quality of energy use in solar still systems.

### Exergoeconomic analysis

**Fig. 29:**
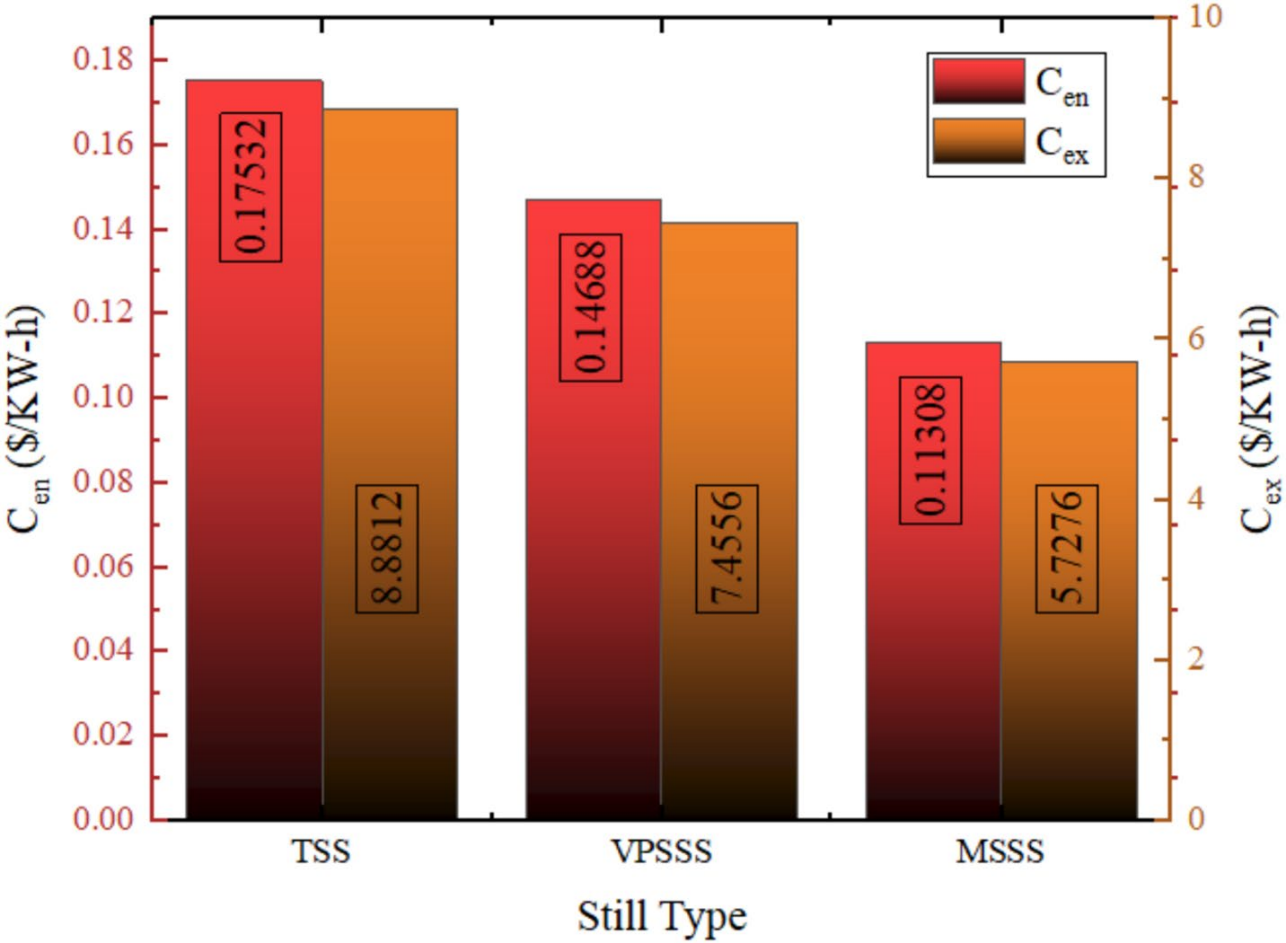
Exergy and energy costs for each Still.

Figure 29 provides the exergy and energy cost of each solar still configuration and the lowest energy cost is of the MSSS (melamine sponge) at 0.075 $/kWh which is then followed by the VPSSS (pumice stone) at 0.098 $/kWh and finally the TSS (traditional) at 0.117 $/kWh. Exergy cost of all systems is more expensive than others, 0.15 $/kWh in TSS, 0.12 $/kWh in VPSSS and 0.097 $/kWh in MSSS because the quality (usability) of thermal energy in distillation is lower than the total energy amount spent. Their cost-effectiveness is due to the fact that MSSS and VPSSS have higher levels of thermal performance: melamine sponge has high porosity (> 99%) and low thermal conductivity (~ 0.035 W/m·K), which allows it to evaporate quickly, whereas pumice stone has moderate levels of porosity (~ 65%) and thermal mass which helps it to retain heat and distribute capillary water, respectively, leading to higher daily productivity and, accordingly, reducing energy and exergy costs per unit output. Lower exergy costs are associated with increased thermodynamic usability; the lowest cost of MSSS of 0.097 $/kWh is due to its high rate of evaporation and the ability to convert the solar heat into usable work potential despite low-grade thermal energy.

**Fig. 30:**
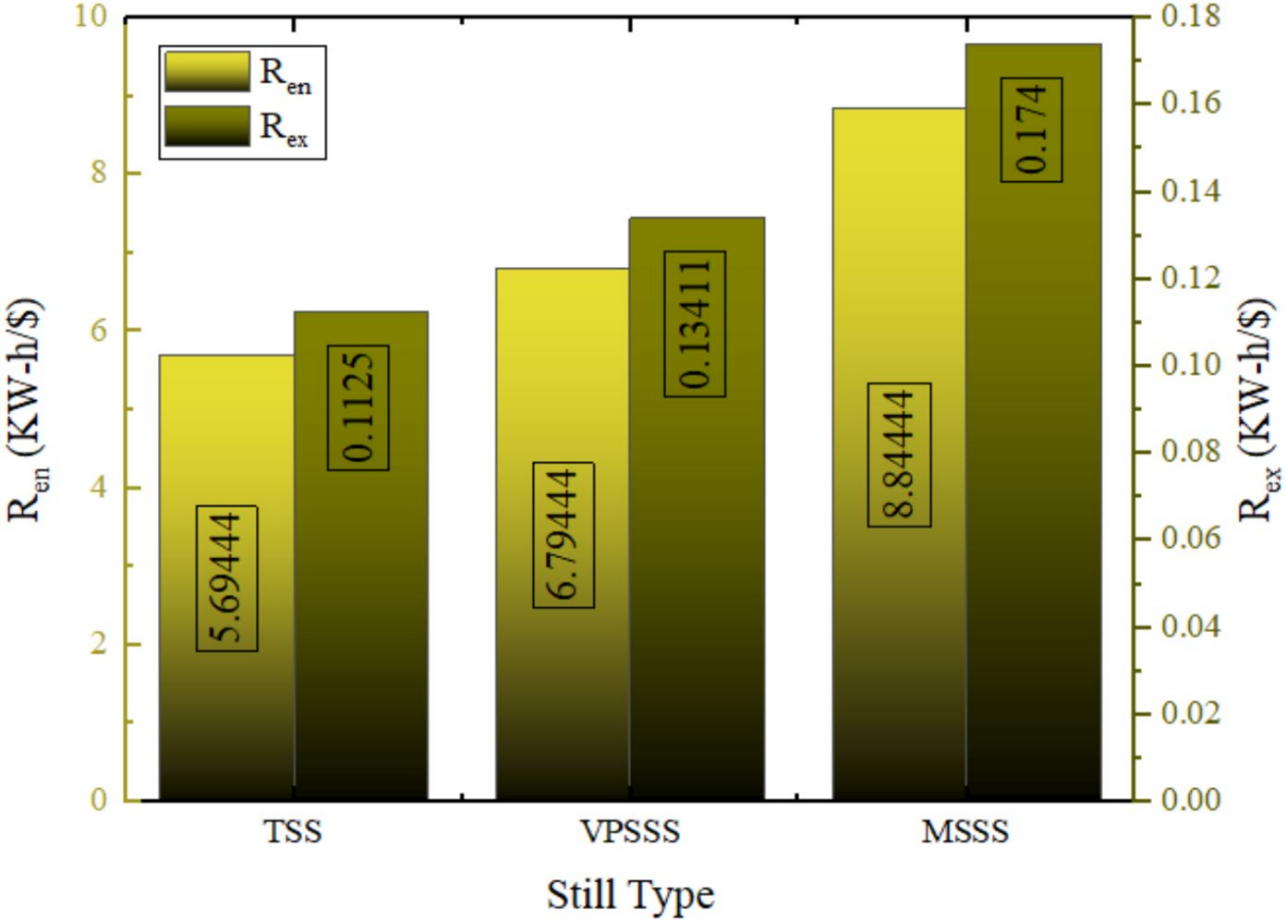
Value of R_en_ and R_ex_ for the three Still.

The energy-economic (Ren) and exergy-economic (Rex) throughput indexes of solar stills of three configurations TSS, VPSSS and MSSS are presented in Fig. 30. The generated useful energy per dollar used is the 5.7 kWh/$ (Ren TSS) with a 19.5% and 55% increase in the values of VPSSS and MSSS, alike, to 6.8 kW-h/$ and 8.8 kWh/$, respectively. They have improved with higher thermal absorption and faster evaporation rates that the porous materials (melamine sponge and pumice stone) provide that raise temperatures of water and, therefore, daily productivity. Unlike that, the values of Rex (useful exergy per dollar) are much lower in all systems since exergy represents the quality and work potential of energy, and the low thermal energy of the solar stills can be converted to useful work only to a limited extent. The better use of incident solar energy by the higher Rex modified stills, but the exergy efficiency in general is lower, which puts the thermodynamic constraints of low-grade thermal desalination processes in perspective.

### Enviro-economic analysis

**Fig. 31:**
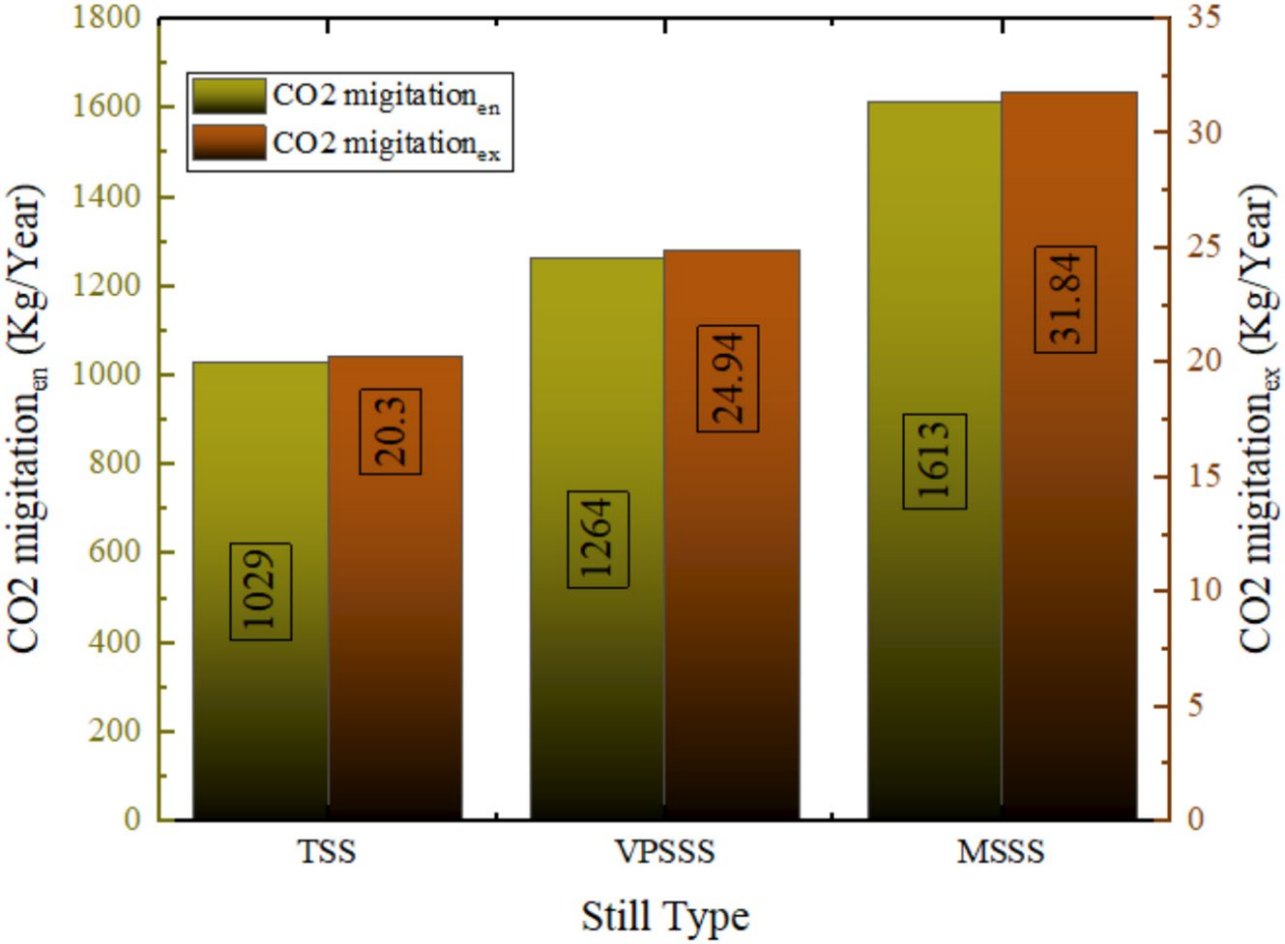
Value of carbon dioxide emissions avoided using the three solar still.

Figure 31 demonstrates that the carbon dioxide emissions were 1029 kg avoided annually using the solar still, which corresponds to the traditional still (TSS), with the pumice stone (VPSSS) and the melamine sponge (MSSS) stills respectively. Such improvements of 22.8% in VPSSS and 56.75% in MSSS are directly related to the thermal performance of the modified stills since even the higher water temperatures and higher evaporation rate with porous materials (particularly, high-porosity melamine sponge) lead to a higher annual production of thermal energy. As this thermal output replaces electricity which would otherwise be produced using fossil fuels (whose CO_2_ emission factor is 2 kg/kWh) the better performance of VPSSS and MSSS results in an equivalent effort to avoid CO_2_ emissions. Higher productivity rate of MSSS (457.9 L/year) had a direct proportional relationship with its avoided CO2 (1612 kg/year), with a greater proportion of fossil-based electricity that was used to run thermal desalination aging out in line with the solar energy input.

**Fig. 32:**
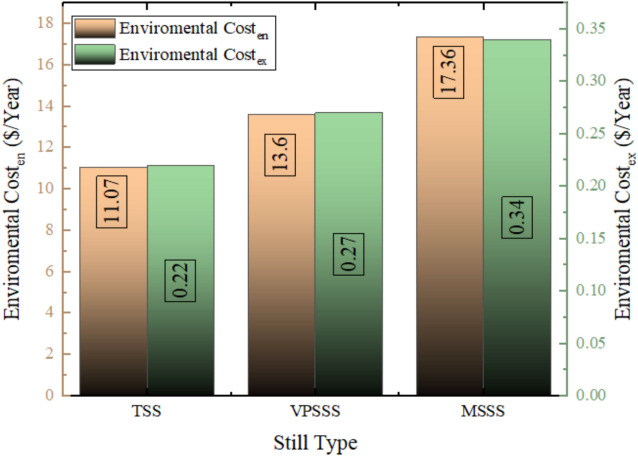
The environmental cost avoided because of using solar still in the three system.

Figure 32 demonstrates that the three solar still systems will avoid the environmental cost as shown below, 22.8% increase in the pumice-enhanced still (VPSSS) and 56.75% increase in the melamine-sponge-enhanced still (MSSS), by savings of 11.07 $/year (traditional still TSS), 13.60 $/year (pumice-enhanced still VPSSS), and 17.36 $/year (melamine-sponge-enhanced still Such values are associated with the shunned CO_2_ emissions caused by replacing electricity that uses fossil fuels with solar thermal power in the desalination process. The increased savings of MSSS and VPSSS can be directly related to their increased productivity because they have a high productivity, which can be attributed to the greater evaporation owing to the greater surface area (melamine sponge) that increases the amount of distilled water produced per unit of solar input and improved thermal retention (pumice stone) which results in more distilled water per unit of the solar input, thus compensating more costly traditional methods of water production. The higher productivity is directly related to the higher saved, as the more surface area the MSSS has to evaporation means the more distant water per watt of sunlight it produces, which is at the expense of the carbon-intensive traditional production methods.

### Comparison with previous literatures

As indicated in the tabular table, our enhanced still (with melamine sponge), MSSS, has a daily productivity of 1347 mL/day and a thermal efficiency of 49.3%, which is higher than some of the latest research using wick media^3^, other porous absorber material^8^, vibrating enhancement^13^, or nanoparticle coating^21^. Moreover, the price per liter of MSSS is extremely competitive as it is only 0.0757$/L, which highlights not only high productivity but also better economic feasibility. All these comparisons validate that the high-porosity low-conductivity melamine sponge integration is an exciting development in the solar still performance, and presents a very attractive balance between high yield, high efficiency and low costs in the small-scale desalination. The comparison of the current work with literatures is presented in Table 3.

**Table 3:** Comparative summary of key performance for present work with literatures.

Reference (Year)	Modification/Enhancement Method	Daily Productivity (mL/day)	Thermal Efficiency (%)	Cost per Liter (USD/L)	Important note
Present study (2026)	Melamine sponge (MSSS)	1347	49.3	0.0757	Best performer in present work
Present study (2026)	Pumice stone (VPSSS)	1055	38.2	0.0985	Significant improvement over TSS
Kadhim et al.^3^ (2025)	Wick materials in single-slope still	~ 950–1150	~ 38–45	~ 0.085–0.110	Wick materials showed notable gains
Ramzy et al.^8^ (2023)	Various porous absorbing materials	~ 600–900	~ 30–40	~ 0.10–0.15	Material choice critically impacts output
Aghakhani et al.^13^ (2025)	Vibrating wire mesh with porous media	~ 1100	~ 42	N/A	Mechanical vibration further boosts yield
Panchal et al.^21^ (2021)	MnO_2_ nanoparticle-coated absorber	~ 980	~ 41	N/A	Nanocoating enhances absorption

## Conclusions

The paper presented an experimental and numerical analysis to improve the productivity, thermal efficiency and economic feasibility of single-slope solar still with porous absorbing materials. The efficiency of a conventional solar still (TSS) was compared to two modified versions, (one with addition of melamine sponge (MSSS) and the other one with pumice stone (VPSSS). Thermal, economic, energy, exergy, and environmental analyses were conducted in order to determine their viability in small scale desalination of arid areas. The highlights, difficulties, and suggestions are as follows:

1) Addition of porous materials greatly enhanced daily freshwater production with the melamine sponge (MSSS) being the most productive (1347 mL/day, 56.9% better) and thermally efficient (49.3%).

2) Economic consideration proved the MSSS to be the most economic choice (0.07569$/L) and the one with the shortest payback period (2.5 years).

3) Energy payback was positive (0.55–0.86 years) however, exergy recovery were still prolonged (28–35 years) and this is a thermodynamic limitation.

4) The environmental advantages were quite high, as the MSSS saved 1612 kg of CO_2_ per year.

5) Experimental findings were confirmed to a high degree (2.36% mean error) by numerical simulations.

6.Future directions: Research should be carried out on hybrid systems that combine phase change materials and porous materials or nanofluids to increase operational times; research into durable, cost-effective, and locally-obtained porous materials to achieve great sustainability; long-term reliability and fouling studies to be performed under continuous saline exposure; and photovoltaic or thermal collector systems to be integrated to increase exergy efficiency and system flexibility.

All in all, it was found that melamine sponge-enhanced solar still (MSSS) was the most promising arrangement, which has the highest productivity, thermal efficiency, and economic payoff with the lowest payback period and hence highly suggestive to adopt in small-scale, off-grid desalination of sunny arid areas like Karbala, Iraq; to scale to wider applicability, future designs need to pay more attention to exergy efficiency, longevity of materials, and adjust systems to changing climate conditions.

## Data Availability

The data supporting the findings of this study can be accessed from corresponding author upon reasonable request.

## Abbreviations

A: Distiller surface area (m^2^)
AAP: Average Annual Productivity (Liter/Year)
AC: Annual cost ($/year)
AMC: Annual maintenance cost ($/year)
ASV: Annual salvage value ($/Year)
CF: Year net cash flow ($/year)
CPL: Cost per Liter ($/Liter)
CRF: Capital recovery factor
E_in_: Total embodied energy (MJ)
E_out_: Annual energy produced (MJ/year)
EPBT: Energy payback time (year)
EPF: Energy production factor
FAC: Fixed annual cost ($/year)
FRP: Factor recovery period
i: Interest rate
LCCE: Life cycle conversion efficiency
$$m$$: Total mass of distilled water produced per day (Liter/day)
MSSS: (Refers to solar still with Melamine Sponge)
np: Payback Period (year)
PCC: Primary Capital cost ($)
R: Result (in uncertainty analysis)
Ren: Ratio of useful energy produced to economic cost (kWh/$)
Rex: Ratio of useful exergy produced to economic cost (kWh/$)
S: Salvage value ($)
SFF: Sinking Fund Factor (1/year)
To: Ambient temperature (K)
Ts: Sun surface temperature (K)
TW: Water temperature (K)
TSS: Traditional Solar Still
UR: Overall uncertainty in result R
U_x_: Uncertainty in variable x
VPSSS: (Refers to solar still with Pumice Stone)
Z: Number of years of operation (year)

## Greek letters

ηc: Conversion efficiency from electricity to heat
ψLCCE: Life cycle exergy conversion efficiency
